# Diazotroph Diversity and Nitrogen Fixation in Summer Active Perennial Grasses in a Mediterranean Region Agricultural Soil

**DOI:** 10.3389/fmolb.2019.00115

**Published:** 2019-11-05

**Authors:** Vadakattu V. S. R. Gupta, Bangzhou Zhang, Christopher Ryan Penton, Julian Yu, James M. Tiedje

**Affiliations:** ^1^CSIRO Agriculture and Food, Waite Campus, Urrbrae, SA, Australia; ^2^Institute for Microbial Ecology, School of Medicine, Xiamen University, Xiamen, China; ^3^Center for Microbial Ecology, Michigan State University, East Lansing, MI, United States; ^4^College of Integrative Sciences and Arts, Arizona State University, Mesa, AZ, United States; ^5^Center for Fundamental and Applied Microbiomics, Biodesign Institute, Arizona State University, Tempe, AZ, United States

**Keywords:** diazotrophs, N fixation, endosphere, phyllosphere, *nifH*, perennial grasses

## Abstract

Summer-growing perennial grasses such as *Panicum coloratum* L. cv. Bambatsi (Bambatsi panic), *Chloris gayana* Kunth cv. Katambora (Rhodes grass) and *Digitaria eriantha* Steud. cv. Premier (Premier digit grass) growing in the poor fertility sandy soils in the Mediterranean regions of southern Australia and western Australia mainly depend upon soil N and biological N inputs through diazotrophic (free living or associative) N fixation. We investigated the community composition and diversity (*nifH*-amplicon sequencing), abundance (qPCR) and functional capacity (^15^N incubation assay) of the endophytic diazotrophic community in the below and above ground plant parts of field grown and unfertilized grasses. Results showed a diverse and abundant diazotrophic community inside plant both above and below-ground and there was a distinct diazotrophic assemblage in the different plant parts in all the three grasses. There was a limited difference in the diversity between leaves, stems and roots except that *Panicum* grass roots harbored greater species richness. Nitrogen fixation potentials ranged between 0.24 and 5.9 mg N kg^−1^ day^−1^ and N fixation capacity was found in both the above and below ground plant parts. Results confirmed previous reports of plant species-based variation and that Alpha-Proteobacteria were the dominant group of *nifH*-harboring taxa both in the belowground and aboveground parts of the three grass species. Results also showed a well-structured *nifH*-harboring community in all plant parts, an example for a functional endophytic community. Overall, the variation in the number and identity of module hubs and connectors among the different plant parts suggests that co-occurrence patterns within the nifH-harboring community specific to individual compartments and local environments of the niches within each plant part may dictate the overall composition of diazotrophs within a plant.

## Introduction

In both agricultural and natural ecosystems, nitrogen (N) is one of the major nutrientss essential for plant growth. While fertilizer addition is an important source of N to agricultural crops, biological processes that supply N, such as biological N fixation, can contribute to more than half of total crop N demand (Angus and Grace, 2017). Within un-disturbed terrestrial ecosystems and perennial grasslands, biological N fixation (BNF) is typically the primary source of N to plants. Global estimates for total terrestrial BNF of 128 Tg N y^−1^ (Vitousek et al., 2013) and 4–55 Tg N yr^−1^ for cropping systems have been reported previously (Herridge et al., 2008). Biological N fixation refers to the metabolically intensive microbial conversion of atmospheric N_2_ to ammonia (NH_3_). This process is mediated by both symbiotic and non-symbiotic microbes including both bacterial (autotrophic and heterotrophic) and archaeal taxa (Gaby and Buckley, 2011). Unlike symbiotic bacteria (e.g., Rhizobia) which fix N_2_ inside nodules, non-symbiotic (NS) N_2_ fixing microbes, which are generally referred as diazotrophs, are identified as free-living within soil or associated with plant roots.

Both free-living and associative N_2_ fixation occurs in association with cereal crops, grasses (including winter and summer active perennial species), sugarcane and other non-leguminous plants (Kirchhof et al., 1997; Roper and Gupta, 2016; Liang et al., 2019). It has also been shown to occur in microsites associated with decomposing plant residues, in aggregates containing decomposable particulate organic matter and in termite habitats (Okuma, 2007; Roper and Gupta, 2016). Perennial grasses generally depend on N mineralized from soil organic matter, atmospheric deposition and biological inputs to meet their N requirements, especially in systems that do not receive anthropogenic (fertilizer) N inputs. While perennial grasses can respond well to the addition of fertilizer N, economic and environmental considerations may preclude fertilizer application for growth in low productivity areas. Therefore, diazotrophic N_2_ fixation can be an important source of N to meet the annual N demand of 20–80 kg N/ha/year by these perennial grasses grown in the Mediterranean regions of southern and Western Australia. Nitrogen fixation by rhizosphere free-living and associative diazotrophs as well as by endophytes in perennial grasses has been well-documented, although the contribution of fixed N to the total grass N requirement and the range of diazotoph diversity and composition remain poorly understood (Zehr et al., 2003; Bahulikar et al., 2014; Roper and Gupta, 2016; Roley et al., 2018).

The warm season perennial grass, switch grass (*Panicum virgatum* L.), a C4 grass in tallgrass prairies of North America, that received recent attention for its biofuel production potential. It has been shown to host diverse diazotrophic communities (Bahulikar et al., 2014; Roley et al., 2019). Within the drier, mixed farming regions of southern Australia, perennial pasture grasses such as *Panicum* spp., Rhodes grass and *Digitaria* species are recommended for both economic and environmental sustainability as they provide stable production throughout the year compared to annual species (Descheemaeker et al., 2014). Due to their extensive root systems (Sanderman et al., 2013), perennial grasses grown during the summer in southern and western Australia contain dense, N-limited rhizospheres that supply significant inputs of C through rhizodeposition contributing to deep soil C inputs and providing a C-rich environment for the maintenance of diazotrophic metabolism. For example, *Panicum* species has been shown to allocate a large portion of photosynthetically fixed C belowground that may be assimilated into the microbial component within a short-period of time (Roper et al., 2013). Compared to cropped soils, it has been suggested that the combination of higher total rhizosphere volume, increased C input, and a greater diversity of rhizosphere bacteria results in significant impacts to N-cycling processes such as N mineralization, with a further impact on soil C turnover (Roper et al., 2013; Sanderman et al., 2013; Gupta et al., 2014). Diazotrophic N_2_ fixation by roots of *Panicum* spp., Rhodes grass and *Digitaria* was reported to range between 0.92 and 2.35 mg ^15^N/kg root/day (Gupta et al., 2014). Reis et al. (2001) reported that grass species *Urochloa brizantha* and *Panicum maximum* obtain up to 41% of their N through biological N_2_ fixation and suggested that this is achieved by allocating large quantities of C through root exudates.

Until recently, the diversity of diazotrophic communities was mostly based on cultivated members of the Proteobacteria, green sulfur Bacteria, Cyanobacteria, and Firmicutes (Roper and Gupta, 2016). Conversely, molecular approaches for characterizing the N-fixing microbial community target the catalyst for this process, the nitrogenase enzyme, which is widely distributed among prokaryotic phyla (Gaby and Buckley, 2011). The nitrogenase enzyme complex consists of two multi-subunit metallo-proteins encoded by the *nifH, nif* D and *nif* K genes, with *nifH* gene generally used as the marker gene (Zehr et al., 2003; Gaby and Buckley, 2011). The *nifH* gene, which encodes the iron nitrogenase unit of the nitrogenase complex, is considered advantageous over others due to a well-conserved amino acid sequence and the presence of extensive reference sequence databases (Zehr et al., 2003; Izquierdo and Nusslein, 2006; Penton et al., 2016). Cultivation independent approaches utilizing this target gene have indicated a high level of diversity in agricultural, forest, rhizosphere and other soil environments (Zehr et al., 2003; Buckley et al., 2007; Kumar et al., 2017) as well as in plants (Hsu and Buckley, 2009). It has been suggested that the varying growth requirements of the phylogenetically heterogeneous diazotrophs may have precluded the cultivation of a substantial proportion of these bacteria. Therefore, it is not surprising that molecular approaches have revealed the presence of a wide diversity of uncultured diazotrophs (Lovell et al., 2000; Buckley et al., 2007).

Research on diazotrophic N fixation has been principally concentrated on bulk and rhizosphere soils, plant residues and roots. Since plant roots are colonized by a subset of microorganisms recruited from the bulk soil, soil characteristics influence the composition of the rhizosphere microbial community (Regan et al., 2014; Bulgarelli et al., 2015), as does plant type (Bulgarelli et al., 2013). For example, a significant effect of plant species and varieties on diazotroph abundance and the number of *nifH* transcripts per root system has been reported (Gupta and Hicks, 2011; Bouffaud et al., 2016). Diazotrophic bacteria have also been found in the above-ground biomass of many plants (Kirchhof et al., 1997; Burbano et al., 2011; Davis et al., 2011; Sessitsch et al., 2012; Moyes et al., 2016) and are thought to live in intercellular spaces in the roots and above-ground biomass without eliciting plant defense responses (Hurek et al., 2002). As these endophytic taxa are uncoupled from the properties of their original inoculation source, the bulk or rhizosphere soil, it is expected that plant properties would exhibit a stronger influence on their composition, abundance, and activities. Indeed, plant host genetic variation has been found to impact the composition of endophytic bacterial and fungal microbiomes (Lundberg et al., 2012; Horton et al., 2014; Bulgarelli et al., 2015; Rodriguez et al., 2019).

Despite there being strong evidence for rhizosphere associated N_2_ fixation with perennial grasses, evidence for the diazotrophic community composition and N_2_ fixation associated with above-ground plant parts in perennial grasses is limited (Chowdhury et al., 2009). For switchgrass (*Panicum virgatum* L.), a warm-season C4 grass grown in Oklahoma and Michigan (U.S.A.), a distinct diazotrophic community associated with the roots and shoots has been identified consisting of members of the Alpha, Beta-, Delta and Gamma Proteobacteria, as well as Firmicutes (Bahulikar et al., 2014; Roley et al., 2019). The most abundant diazotrophic genera identified in switchgrass from Michigan included *Bradyrhizobium, Hyphomicrobium, Geobacter, Polaromonas*, and *Methylocella* (Roley et al., 2019). In contrast, the prominent genera recovered from native grass in the warmer and drier Oklahoma prairie were *Rhizobium, Methylobacterium, Burkholderia, Azoarcus*, and *Geobacter* (Bahulikar et al., 2014). Gupta et al. (2014) reported that different summer active C4-perennial grass species promoted the abundance of specific members of the soil *nif* H community in the roots, suggesting plant-based selection from the soil diazotrophic community. This selection process is supported by the finding that abundance of diazotrophs in the grass rhizosphere soils were similar to populations in the roots but the diversity of diazotrophic bacteria was significantly higher in the rhizosphere than in the roots (Gupta et al., 2014).

Summer active perennial grasses in the Mediterranean regions of southern and western Australia are generally grown in lower fertility soils without significant fertilizer addition. This study investigated the abundance, diversity and functional capability of the diazotrophic microbial community in the below-ground and above-ground biomass of C4-grasses e.g., *Panicum coloratum* L. cv. Bambatsi (Panicum), *Chloris gayana* Kunth cv. Katambora (Rhodes grass), and *Digitaria eriantha* Steud. cv. Premier (Digitaria) grasses grown on a sandy loam soil in South Australia. To accomplish this, functional gene (*nifH*) amplicon sequencing and quantitative PCR were used to determine the diversity and abundance of the diazotroph (*nifH* gene harboring) community coupled with the determination of N_2_-fixation potential by ^15^N-enriched *in-vitro* assays.

## Materials and Methods

### Field Site and Sampling

The field experimental site is located at Karoonda, South Australia (35°05′S, 140°06′E) in a Mediterranean climate. Details of soil properties and climate are provided in Table S1. Briefly, the climate at the site is characterized by a winter dominant rainfall with an average monthly rainfall of 28.4 ± 5.3 mm, annual maximum temperature (MAT) 30.2 ± 4.1°C and minimum temperature (MIT) of 12.5 ± 2.1°C. The summer active perennial grasses investigated in this study were established in 2010 and generally grow between December and April. The perennial grasses did not receive any chemical fertilizers during the experiment. Soil type is a Calcarosol (Gupta et al., 2014) and sandy loam in texture (6% clay and 75% sand) with pH (water) 6.35, organic C 1.04%, and total N of 0.08%. Mineral N levels in the surface soil at the time of sampling were <5 ppm.

We investigated diazotrophic (*nifH* gene harboring) microbial communities associated with above- and below-ground plant parts of three different summer active perennial grasses. Plant samples for microbial and N_2_ fixation measurements were collected from the field experiment when grasses were actively growing during the summer (March) of 2014. Roots and above-ground plant material collected from five plants in three replicate field plots were brought to the lab where the leaf and stem parts were separated. Roots were initially washed with distilled water to remove adhered soil. All plant parts were surface sterilized following the procedure of 1 min 80% ethanol followed by 1 min 4% NaOCl and 1 min 80% ethanol and finally 3 rinses in sterile DW. Subsamples of the surface sterilized samples were freeze-dried prior to DNA extraction or directly used in the ^15^N_2_ fixation measurement using a 5 day laboratory incubation assay with ^15^N enriched (98% atom) headspace at 25°C (Gupta et al., 2014). Plant samples were also collected during the summer (March) in 2015 for additional ^15^N_2_ fixation laboratory assays.

### DNA Extraction Procedure

DNA was extracted from 0.5 to 1.5 g of freeze-dried root, stem or leaf samples using the DNeasy PowerMax soil kit (www.qiagen.com) following the manufacturer's protocol with modifications including an initial liquid nitrogen homogenization of the plant material using the red rock sand from the PowerMax Power-bead Falcon Tube. Further mechanical disruption of the plant material was achieved by placing homogenized material into a shaking water bath at 65°C for 45 min on maximum speed (as per the DNeasy PowerMax method). Final DNA extracts were eluted using 1 ml of warmed (60°C) C6 solution two times (5 min each) to maximize DNA yield (a final volume of 2 ml) and the extracts stored at −80°C. DNA extracts were also further cleaned using MinElute 96 UF PCR Purification Kit (www.qiagen.com.au) and DNA eluted into nuclease free water.

### Enumeration of Total Bacteria and N_2_-Fixing (Diazotrophic) Bacteria

DNA in each sample was quantified against a DNA standard (λ-phage DNA; *R*^2^ = 0.98) using the QuantiT PicoGreen dsDNA assay (Invitrogen, MA, USA). The final extracted DNA was diluted 1:10 to a final volume of 50 μL in molecular grade H_2_O and 3 μL was used per 15 μL PCR reaction. Total bacterial abundances were quantified using primers F968 /R1378 (Smalla et al., 2007), based on the chemistry of the QuantiTect SYBR Green PCR kit (Qiagen, Vic, Australia). Amplification was carried out on a Strategene Maxpro3000P qPCR system (Agilent, Vic, Australia). qPCR was performed against a standard curve of known 16S rRNA gene copies generated using a pGEM-Teasy vector cloned standard (Gupta et al., 2014). Abundances of the *nifH* gene was measured by using specific functional gene primers *nifH*-F and *nifH*-R (Röesch et al., 2008; synthesized by Geneworks Pty Ltd, Adelaide, S. Aust.), based on the QuantiTect SYBR® Green PCR kit. Amplification was carried out on a Maxpro3000P qPCR system. To confirm the specificity of PCR products, all PCR reactions were followed by melting curve analysis and agarose gel electrophoresis. A standard curve containing known copy numbers of the *nifH* gene was generated using 10-fold serial dilutions (10^1^–10^6^ gene copies) of linearised plasmids containing a cloned *nifH* gene from pure cultures of *Azospirillum* sp. generated by using the pGEM-T Easy vector and Wizard® Plus SV Miniprep DNA Purification system, according to the manufacturer's instructions (Promega Inc., Fitchburg, WI, USA).

*NifH* gene amplification for sequencing was performed using the PolF (TGCGAYCCSAARGCBGACTC) and PolR (ATSGCCATCATYTCRCCGGA) primers using previously described methods (Zhang et al., 2015). Briefly, PCR mixtures contained 1x green buffer (Promega, Madison, WI), 1.8 mM MgCl_2_ (Promega), 0.5 mM each deoxynucleoside triphosphate (Promega), 500 nM primer (IDT), 0.1 mg/ml bovine serum albumin (NEB), 2.5 U of Taq polymerase (Promega), and 1 ng/ul template DNA in a 20-ul reaction volume. Amplifications were performed for 3 min at 95°C, 30 cycles of 45 s at 94°C, 45 s at 61°C, and 1 min at 72°C, followed by a final extension for 7 min at 72°C. Following gel extraction using the QIAquick gel extraction kit (Qiagen), the eluted DNA was further purified again using the Qiagen PCR Purification kit (Qiagen). DNA samples were amplified with adaptors for Ion Torrent sequencing at the 5′ end, followed by an 8 bp barcode to separate samples (Poly et al., 2001; Zhang et al., 2015). Sequencing of the 24 samples was carried out using the Ion Torrent PGM system using chip 318 v2 with the 400 bp Sequencing Kit in the University of Wisconsin Biotechnology Center.

The FunGene pipeline (http://fungene.cme.msu.edu/FunGenePipeline/) was used to process the sequence results using the parameters described by Fish et al. (2013) and Zhang et al. (2015). Raw sequencing data were trimmed by RDP Initial Process which produced a total of 406, 973 full-length high-quality reads. After chimera check and frameshift correction, 402,537 reads were retained and clustered at 5% amino acid dissimilarity by the Fungene pipeline. NifH clusters and closest matched taxa were obtained at the frameshift correction step by FrameBot (Wang et al., 2013). All samples were subsampled to 9,059 reads/sample for diversity calculation and ordination. Data were plotted in R 3.1.3 with packages of vegan and ggplot2. Venn diagrams were generated to assess the distinct and common NifH phylotypes (OTUs) among different plant parts using the criteria of OTU occurrence in at least two replicate samples for each plant part. Rarefaction curves were constructed using the “-fasta_rarify” command in Usearch (v8). The method used to calculate these curves is described at http://drive5.com/usearch/manual8.1/rare.html. The OTU tables for each of the three sample types were first converted to size-annotated FASTA sequences files and each these files were then processed using Usearch to generate the data for the rarefaction curves.

### Multivariate, Neighbor Joining Tree, and Diversity Analyses

Raw cluster abundances were Hellinger transformed and a Bray-Curtis dissimilarity matrix (+1) was constructed. Statistical analyses and diversity estimates were calculated using PRIMER-E (Primer 7, Clarke and Gorley, 2006). Cluster analysis was performed with the Similarity Profile analysis (SIMPROF) test (Clarke et al., 2008). Significant differences in community structure were tested for different grass species and plant parts with Permutational Multivariate Analysis of Variance (PERMANOVA) (Anderson, 2001) and Analysis of Similarity (ANOSIM) (Clarke and Ainsworth, 1993). Additionally, OTU abundance data were Hellinger transformed and subjected to redundancy analysis (RDA) for ordination under constraint of the plant parts. Canonical analysis of principle coordinates (CAP) analysis was performed for different plant parts found to be significant according to PERMANOVA analysis. ANOVA statistics for Shannon diversity (H), Pielou's Evenness (J), Margalef's Richness (d) and the Simpson index were performed using GENSTAT version 8 (VSN International Ltd, Hemel Hempstead, UK). A neighbor-joining tree, based on aligned amino acid sequences, was constructed using the top 134 most abundant OTUs and visualized in iTOL (Letunic and Bork, 2011). OTU representative sequences were assigned to their closest phylogenetic match by BLASTp against an updated Zehr-based NifH database with the protein region corresponding to the primer coverage extracted, based on the original dataset previously documented (augmented Zehr-set, Wang et al., 2013). Assignment to Zehr clusters was based on the ARB database (Heller et al., 2014).

For analyses of phylogenetic structure, the community data was filtered to match the taxa included in the neighbor-joining tree using the function match.phylo.comm in R studio. To evaluate phylogenetic signal across the different plant parts, we first found the niche values, the abundance-weighted mean of the environmental variable, for plant-C and plant-N for each OTU (Table S6). The between-OTU niche differences were related to phylogenetic distances calculated with Mantel correlograms with 999 randomization for significance test with the function “mantel.correlog” in the package Vegan v2.4.1 (Oksanen et al., 2018). Mantel tests showed no significant correlations across phylogenetic distances, except a significant negative correlation at 0.003 of the phylogenetic distance (Figure S6). Thus, we did not calculate phylogenetic turnover and instead determined the phylogenetic community composition within each sample. To characterize phylogenetic community composition, net relatedness (NRI) and nearest taxon index (NTI) were calculated using the “ses.mpd” and “ses.mntd” functions in the “picante” package (Kembel et al., 2010). These indices describe the degree to which phylogenetic structure of samples show significant evenness or clustering. Positive NTI and NRI values indicate the community is phylogenetically clustered while negative values indicate that members of the community are phylogenetically randomly dispersed. For a community, NTI >2 indicates phylogenetic clustering and NTI < −2 indicates phylogenetic dispersion. A mean NTI and NRI across all samples that is significantly different from zero indicates clustering (>0) or overdispersion (<0) (Kembel, 2009; Ding et al., 2012; Stegen et al., 2012). We used a Wilcoxon test to determine whether mean NRI and NTI were significantly different (*p* < 0.05) from zero.

### Molecular Network Analysis

To decipher the diazotroph community co-occurrence patterns, molecular ecological networks for the *nifH*-harboring bacterial communities were constructed based on NifH sequences, the OTU table was filtered to remove singletons and doubletons. Individual networks were constructed for leaf, stem and root samples across all three plant types since PERMANOVA analysis showed no significant difference between the three plant types. Networks were constructed using the Molecular Ecological Network Analysis (MENA) Pipeline (http://ieg4.rccc.ou.edu/MENA/login.cgi) (Deng et al., 2012). Prior to network construction the appropriate similarity threshold was identified using random matrix theory implemented in the MENA pipeline. The resulting networks were visualized using Cytoscape 3.4.0 (Shannon et al., 2003).

Detection of modules and module calculations were carried out using the greedy modularity optimization method (Deng et al., 2012). A modularity index threshold of 0.4 was used to define modular structures in the network (Newman, 2006). Identification of node topology within the networks was determined based on its within-module connectivity (*Zi*) and among-module connectivity (*Pi*), which was then used to organize nodes into four categories: network hubs (Zi > 2.5, Pi > 0.62), module hubs (Zi > 2.5, Pi < 0.62), connectors (Zi < 2.5, Pi > 0.62), and peripheral nodes (Zi <2.5, Pi <0.62) (Olesen et al., 2007; Zhou et al., 2010; Deng et al., 2012).

### Statistical Analyses

All results were analyzed for normality of residuals in GENSTAT version 8 (VSN International Ltd, Hemel Hempstead, UK), and the statistical significance of treatment differences was tested with analysis of variance at P = 0.05. Data for 16S rRNA gene and *nifH* gene abundances from qPCR analysis were log-transformed before ANOVA test for treatment effects.

## Results

### System Description in Terms of Soil Properties and Grass Growth Patterns for 2014 and 2015 Including Belowground Biomass Data

**Figure 1:**
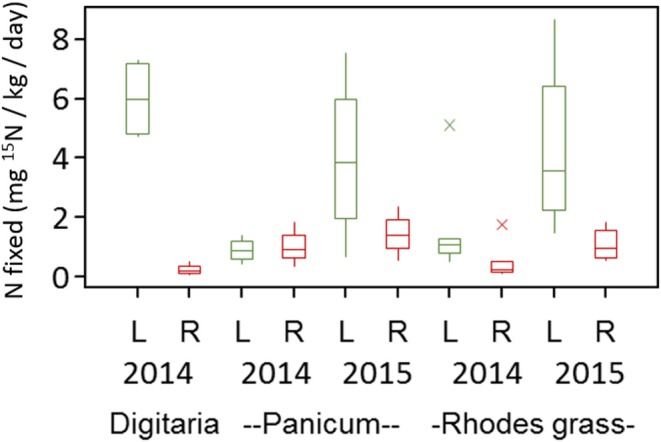
Nitrogen fixation potential for the leaf (L) and root (R) samples of different perennial grasses estimated using a laboratory ^15^N-incubation assay. Based on minimum ^δ^15N (%_o_) value for detection of N_2_ fixation, the minimum detectable N fixation rate was 0.10 mg N fixed kg^−1^ day^−1^.

Under the Mediterranean climate at Karoonda in the mallee region of South Australia, the three perennial grasses produced 0.4–1.5 t/ha of plant biomass. The total amount of biomass produced was higher during the summer of 2014 (0.8–1.5 t/ha) than the summer of 2015 (0.4–0.8 t/ha). This was principally due to the amount and distribution of rainfall, however all plants were in their peak biomass production period during the time of our sample collection (March) (Table S1). *Panicum* species produced the highest amount of biomass in both seasons and the Rhodes grass produced similar amount of biomass in both seasons (Table S1). Soil mineral N levels were generally low (<5 μg/g) during the summer months in both seasons, reflecting the lower fertility from the low organic C levels (Table S1). Overall, at the end of the summer growth period the three grass species removed 30–60 kg N / ha in the two seasons. Nitrogen fixation was observed in all grasses and plant parts. Nitrogen fixation potential estimates measured using a laboratory ^15^N incubation assay indicated rates ranging from 0.24 to 5.9 mg of ^15^N fixed kg^−1^ plant material day^−1^ (Figure 1). While estimates varied between seasons, measurable N_2_ fixation rates were observed in both the leaf (2.13 to 5.99 ± 0.70 mg of ^15^N fixed kg^−1^ plant material day^−1^) and root (0.24 to 2.73 ± 0.18 mg of ^15^N fixed kg^−1^ plant material day^−1^) sample in both seasons (Figure 1). Nitrogen fixation rates also showed small variation between grass types i.e., Digitaria (3.21 ± 0.71 mg of ^15^N fixed kg^−1^ plant material day^−1^), *Panicum* (1.79 ± 0.40 mg of ^15^N fixed kg^−1^ plant material day^−1^), and Rhodes grass (2.10 ± 0.47 mg of ^15^N fixed kg^−1^ plant material day^−1^). Nitrogen fixation was also observed in the crowns of Digitaria grass (1.21 ± 0.42 mg of ^15^N fixed kg^−1^ plant material day^−1^).

### Abundance of Diazotrophic Bacteria

Significant differences in the total bacterial abundance, based on 16S rRNA gene copy numbers, were observed between the three grass species and between the above and belowground plant parts (Table 1). Overall bacterial abundance was significantly higher in the *Digitaria* and *Panicum*, compared to the Rhodes grass. In all three grasses, bacterial abundance was significantly higher in the stems and leaves compared to the endophytic root environment (Table 1). Stem samples from *Panicum* and Rhodes grasses exhibited the highest bacterial total abundance, whereas there was no significant difference between the leaves and stems of *Digitaria* (Table 1). The abundance of diazotrophic bacteria, based on *nifH* gene copy numbers, ranged between 8.4 × 10^5^ to 1.2 × 10^7^ g^−1^ plant material with significant differences occurring between grass species and plant parts (Table 1). *NifH* gene abundance was highest in the *Digitaria* plant samples followed Rhodes grass and *Panicum*. This trend was observed in both the absolute *nifH* gene abundances and in copy number per unit 16S rRNA gene copies. In general, root samples for all the grass species exhibited the highest *nifH* gene abundance (5.44 × 10^5^ to 1.2 × 10^7^) compared to the stem (1.5 × 10^6^ to 3.8 × 10^6^) and leaf samples (8.4 × 10^5^ to 1.8 × 10^6^) (Table 1). Similarly, the proportion of diazotrophic bacteria (*nifH* copy number) to total bacteria (16S rRNA gene copy number) was generally higher in the root samples compared to the above-ground plant parts with no significant difference between the stems and leaves.

**Table 1:** Abundance of total bacteria and N_2_-fixing (*nif* H-harboring) bacteria in different parts for the three types of grass species.

		**Bacteria (16S)**	**N2 fixers (*****ni*****fH)**	
**Plant type**	**Plant part**	**copies/g soil**	**copies/g soil**	**(copies/105-16S) Digitaria**
Digitaria	Roots	3.21E+09	1.17E+07	342.7
	Stems	3.09E+10	1.47E+06	5.3
	Leaves	3.54E+10	1.79E+06	5.8
	**Average**	**2.31E+10**	**5.OOE+06**	**117.9**
Panicum	Roots	2.23E+09	5.36E+05	30.1
	Stems	4.67E+10	3.82E+06	7.9
	Leaves	2.56E+10	8.41E+05	3.6
	**Average**	**2.49E+10**	**1.97E+06**	**13.9**
Rhodes	Roots	3.79E+09	2.21E+06	68.5
	Stems	2.46E+10	2.41E+06	10.5
	Leaves	1.30E+10	1.41E+06	12.8
	**Average**	**1.38E+l0**	**2.01E+06**	**30.6**
Plant parts	Roots	**3.05E+09**	**4.76.E+06**	**147.08**
	Stems	**3.47E+l0**	**2.70.E+06**	**7.89**
	Leaves	**2.25E+l0**	**1.29.E+06**	**7.42**
LSD (*P* <0.05)	Plant type	6.58E+09	2.77E+06	15.95
	Plant part	6.50E+09	2.74E+06	115.5
	Plant × Part	1.14E+10	4.84E+06	202

*Values in bold represent averages for individual plants or combined plant parts*.

### *NifH*-Harboring Bacterial Community Composition

**Figure 2:**
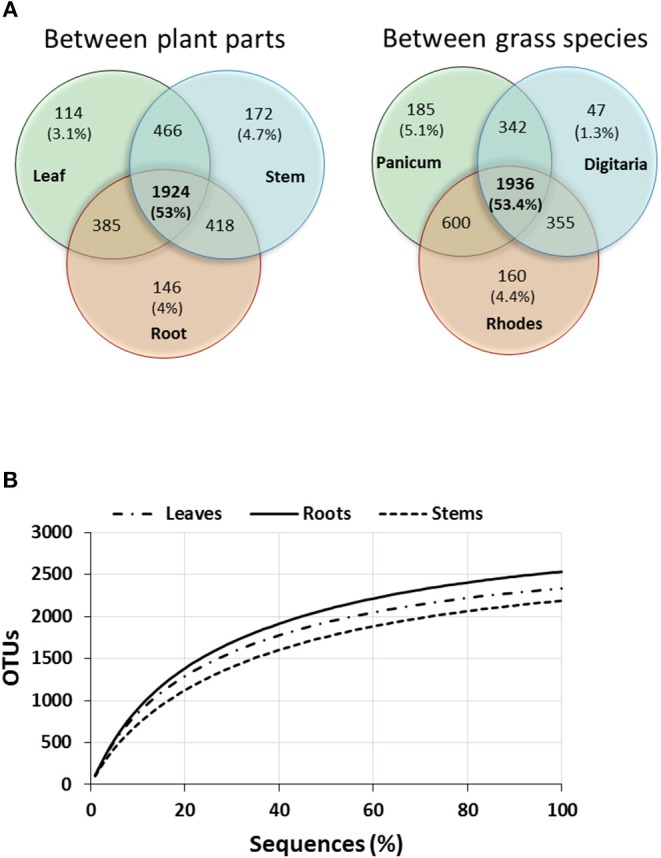
Venn diagram showing number of unique and shared OTUs of total OTUs between different plant parts and grasses **(A)** and rarefaction curves of OTUs of NifH sequences **(B)** cumulatively obtained from the three plant parts (averages for the three perennial grass species).

A total of 402,537 *nifH* amplicon reads were clustered at 5% amino acid dissimilarity by Fungene pipeline. NifH clusters and closest matched taxa were obtained at frameshift correction step by FrameBot and sequences were subsampled to 9,059 reads/sample for diversity calculation and ordination. Rarefaction curves reflected saturation in terms of number of OTUs vs. sequences for all plant parts indicating that the sequencing depth was adequate to cover the majority of the diazotrophic community (Figure 2B). Following clustering and removal of singletons there were 1,067 ± 31, 1,042 ± 34, and 1,007 ± 76 NifH OTUs per sample for the leaf, stem and root samples, respectively. Average protein-protein identity was 93.3 ± 4.1% at an average alignment length of 106 amino acids. A total of 130 unique closest-match taxa were recovered representing 76 unique genera (Table S5).

Significant differences in alpha-diversity were identified between the three grass species (P < 0.01) with *Panicum* exhibiting the highest species richness (d) and evenness (J) (Table 2). No significant differences were observed between *Digitaria* and Rhodes grass (Table 2). There were no significant differences in the overall NifH sequence diversity for the leaf, stem and root samples, although significant differences (*P* < 0.01) were found between the different plant parts for individual grass species. Species richness in the *Digitaria* grass was highest in the leaves whereas roots and stems showed significantly higher richness in the *Panicum* and Rhodes grasses, respectively (Table 2).

**Table 2:** Diversity indices for *nif* H-gene harboring bacterial communities in different plant parts for different grass species.

**Plant type**	**Plant part**	**Species richness (d)**	**Pilou's evenness (j)**	**Shannon index (H)**	**Simpson index (1-**λ)****
Digitaria	Roots	321	0.9540	6.5762	1.0464
	Stems	334	0.9569	6.6578	1.0429
	Leaves	390	0.9659	6.9193	1.0366
	**Average**	**348**	**0.9589**	**6.7178**	**1.0419**
Panicum	Roots	410	0.9652	6.9622	1.0364
	Stems	373	0.9617	6.8223	1.0396
	Leaves	356	0.9609	6.7677	1.0403
	**Average**	**380**	**0.9626**	**6.8507**	**1.0387**
Rhodes	Roots	353	0.9598	6.7499	1.0406
	Stems	372	0.9626	6.8368	1.0383
	Leaves	336	0.9568	6.6690	1.0424
	**Average**	**354**	**0.9597**	**6.7519**	**1.0404**
Plant parts	**Roots**	**361**	**0.9596**	**6.7628**	**1.0411**
	**Stems**	**360**	**0.9604**	**6.7723**	**1.0402**
	**Leaves**	**361**	**0.9612**	**6.7853**	**1.0398**
LSD (*P* <0.05)	Plant type	13	0.0020	0.0548	0.0012
	Plant part	ns	ns	ns	ns
	Plant × Part	23	0.0034	0.0949	0.0021

*Values in bold represent averages for individual plants or combined plant parts*.

**Figure 3:**
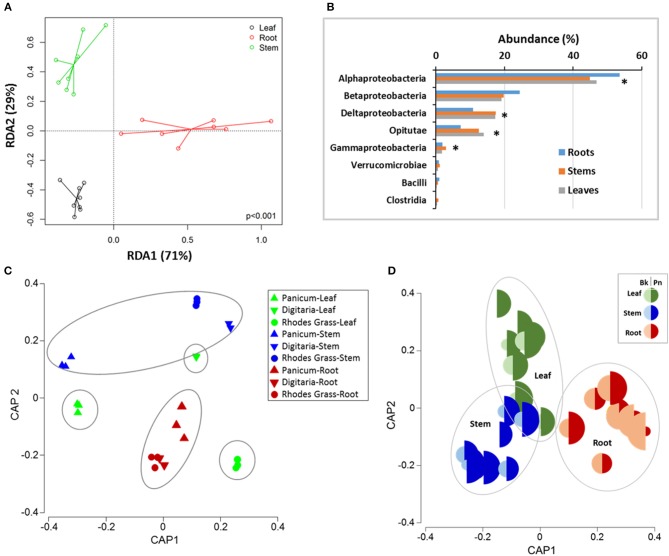
Differences in the composition of *nifH*-gene harboring bacteria in the different above-ground plants parts and roots for the three grasses. **(A)** Redundancy analysis (RDA) for ordination under constraint of the plant parts, **(B)** relative abundances at Class level between different plant parts; ^*^ indicates significant differences at *P* < 0.05, **(C)** Canonical analysis of principle (CAP) ordination, constrained by plant part and species and **(D)** Relative abundance of *Burkholderia* sp (Bk, OTU378) and *Polaromonas naphthalenivorans* (Pn, OTU909) in different plant parts. PERMANOVA—Grass: CV = 5.87, *P* = 0.09; Plant part: CV = 13.7, *P* = 0.01; Grass x plant part: CV = 7.2, *P* = 0.19; Grass-plant part: CV = 13.529; *P* = 0.001; ANOSIM (plant parts): Global R 0.332; *P* = 0.01.

Diazotrophic NifH communities were significantly (PERMANOVA, *P* < 0.001) different among plant parts. Comparison of *nifH*-harboring bacterial community composition from beta-diversity analysis using RDA resulted in significant dissimilarity between the leaf, stem and root samples across all plant types (Figure 3A). PERMANOVA analysis showed that plant part explained 13.7% variation (*F* = 1.91; *P* = 0.001; ANOSIM *P* = 0.01) whereas the variation between grass species was not-significant (*F* = 1.17; CV% = 5.8; *P* = 0.09). Diazotrophic communities within leaves from the three individual grass species were more different than those originating from roots (Figure 3 and Figure S1). The contribution of individual closest-match genera to the overall Bray–Curtis distances (SIMPER analysis) showed that differences in the abundances of 100 OTUs accounted for 15% of the dissimilarity between the root and leaf samples and between the root and stem samples. The sequences most related to Alpha-Proteobacteria contributed to the discrimination between the plant parts. Results from the SIMPER analysis showed that at the OTU level, differences in the diazotrophic communities within leaf and root samples was principally attributed to the variation in the relative abundances of ~20 OTUs representing taxa such as *Hypomicrobium* sp., *Bradyrhizobium* sp., and *Opitutaceae* sp. In contrast, differences between the diazotrophic communities in the leaf and stem samples were associated with differences in the relative abundances of OTUs such as 0487 (*Opitutaceae bacteriuam* TAV2), 0012 (*Dechloromonas aromatica*), 1599 (*Hypomicrobium* sp.), 58 (*Leptothrix cholodnii*) (Figure S3).

**Figure 4:**
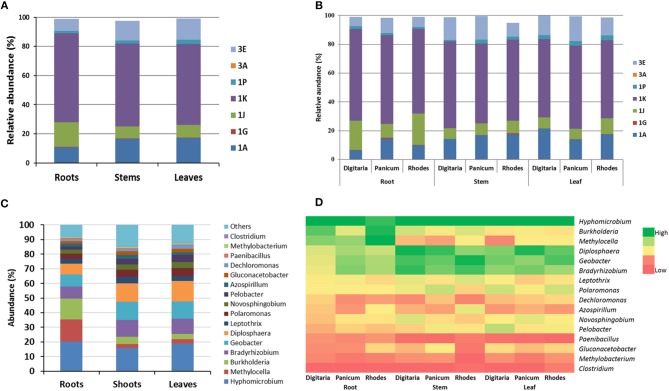
Relative abundances (averages of replicates) of endophytic NifH clusters **(A,B)** and taxa abundances in **(C,D)** different plant parts (leaf, stem and root) for the different summer-active perennial grasses. For clarity only the cluster groups or taxa that showed >0.5% abundance are shown.

In general, *nifH*-gene harboring bacteria most closely related to the phylum Proteobacteria were the most abundant group (Figures 3, 4). Diazotrophic taxa of the Class Alpha-Proteobacteria were generally most dominant (48 ± 1.5%,) followed by Beta-Proteobacteria (21.2 ± 1.25%), Delta- Proteobacteria (15.1 ± 1.5%), and Opitutae (11.1 ± 1.3%) (Figure 3). Abundances of the members of the Verrucomicrobiae were higher in the *Digitaria* root and leaf samples (>1.45%), compared to that in the other grass species (<0.77%).

Diazotroph NifH grouped under Clusters I and III were generally the most abundant group with subcluster 1K being the most abundant (58.1 ± 1.1%) across all plants and plant parts (Figures 4A,B). The abundances of the dominant subclusters were similar in all samples, except that the members of subcluster 1J were significantly (*P* < 0.028) higher in the root samples (16 ± 3.9%), in particular in the roots of *Digitaria* (20.3) and Rhodes grass (21.8%) (Figure 4B). NifH belonging to the subcluster 3E were significantly (*P* < 0.022) more abundant in the above ground plant parts (14.0 and 15.3% in stem and leaf samples, respectively), compared to the root samples (8.1%). NifH belonging to subgroups 1A and 1P were significantly enriched (*P* < 0.05) in the root samples, compared to that in the leaf and stem samples.

The top 10 most abundant genera accounted for ~80% of sequences among all plant parts and grasses. In total, the genus *Hyphomicrobium* like taxa was the most abundant (13.6–29.9%) in all plant parts and grass species) (Figures 4C,D), followed by those related to *Burkholderia* and *Methylocella* in roots, and *Geobacter* and *Diplosphaera* in leaves and stems. Sequences closely related to the genus *Azospirillum* accounted for 1.57–2.94% of the diazotrophic community with higher abundances in the stem samples of Rhodes grass (5.1%). There were significant differences in the relative abundances of *Burkholderia*-like taxa between grass species, especially in the root samples. For example, relative abundances ranged between 18 and 20% in the roots of *Digitaria* and Rhodes grass, compared to 5.8% in *Panicum* roots.

### Phylogenetic Community Structure

To describe the differences between phylogenetic distances in the communities compared to null-communities generated by randomization, NTI and NRI was calculated. The NTI measures tip-level divergences in the phylogeny while NRI measures deeper divergences. For communities across all plant parts and grass species, NTI values were positive but <2 and were significantly different from the null expectation (*p* < 0.05) in most of the communities (Figure S7). NRI values were generally negative and we did not find significant differences from the null expectation (Figure S7). The mean NTI was significantly greater than zero, while the mean NRI was significantly less than zero. These results may indicate that all communities contain species that are spread randomly across the tree (NRI <0) but phylogenetically clustered toward the tips (NTI > 0).

### Network Analysis

To identify co-occurrence patterns in the *nifH*-gene harboring bacterial communities associated with different plant parts, four networks were constructed for the roots, stem, leaf and the whole plant. Individual networks were constructed based on 1,329, 1,307, 1,329, and 1,907 OTUs for the leaf, stem, root and whole-plant, respectively. All networks obtained exhibited scale-free characteristics, as indicated by *R*^2^ of power law ranging from 0.742 to 0.908 and were significantly different from the random networks generated using identical numbers of nodes and links (Tables S2, S3). These metrics indicate that the networks were non-random and unlikely due to chance.

Networks reflecting the three individual plant parts were very similar to one another. However, multiple network topological metrics consistently showed that microbial co-occurrence patterns for the different plant parts were markedly different from the whole-plant network (Table 3). Individual plant part networks were much larger and more complex than the whole-plant network with more links and nodes than the whole-plant network, which increased the density of connections and created more intricate network patterns (Table 3; **Figure 6**; Figure S4). Additionally, the number of modules were greater in the roots, leaf, and stem networks (each composed of 27 modules that contained at least 5 nodes) compared to the whole-plant network (12 modules that contained at least 5 nodes) (**Figure 6**; Figure S4). Interestingly, the whole-plant network contained more modules that were composed of only one taxon at the genus level, which was not observed in the networks of the individual plant parts. Across all networks, the majority of interactions were in the negative direction: 60.5% of 880 links, 70.7% of 1,130 links, 71.5% of 1,143 links, and 62.7% of 351 links in the root, leaf, stem and whole-plant networks, respectively (Tables S2, S3). In contrast to the interaction patterns in the networks of the individual plant parts, negative and positive interactions were generally constrained within individual modules in the whole-plant network (**Figure 6D**).

**Table 3:** Topological properties of molecular ecological networks for *nifH*-gene harboring bacterial communities.

**Network property**	**Roots**		**Leaf**		**Stem**		**All parts**	
RMT Threshold	0.92		0.91		0.91		0.72	
Modularity	0.845		0.908		0.871		0.684	
Total nodes	631		760		723		345	
	1,455		1,599		1,591		560	
**Total links**	**Negative**	**Positive**	**Negative**	**Positive**	**Negative**	**Positive**	**Negative**	**Positive**
	880(60.48%)	575 (39.52%)	1130 (70.67%)	469 (29.33%)	1143 (71.84%)	448 (28.16%)	351 (62.68%)	209 (37.32%)
R2 power-law	0.742		0.744		0.729		0.908	
Average degree (avgK)	4.612		4.208		4.401		3.246	
Ave. clustering coefficient (avg. CC)	0.156		0.141		0.141		0.097	
Harmonic geodestic distance (HD)	5.74		7.90		6.684		4.226	
No. of modules	51		51		45		36	

The topological roles of the specific nodes within the networks were classified according to the Zi vs. Pi coefficients (Table S4; Figure S5) (Zhou et al., 2010). The majority of nodes across all networks were classified as peripheral nodes (Figure S5). The whole-plant network contained one network hub, seven module hubs and seven connectors (Table S3). The network hub was an OTU most closely related to an unidentified bacterium. *Methylocella, Leptothrix and Geobacter* were identified as both module hubs and connectors in the whole-plant network. In addition, *Burkholderia* and *Opitutaceae* were identified as module hubs and *Bradyrhizobium* and *Gluconacetobacter* were connectors in the whole-plant network. In the networks of the individual plant parts, several module hubs and connectors were identified that were unique to either the roots, stems or leaves. For example, module hubs identified as *Herbaspirillum, Leptothrix* and *Methylobacterium* were only observed in the leaf network and the module hub Rubrivivax was unique to the stem network (Table S4). Additionally, connectors identified as *Hyphomicrobium* and *Geobacter* were only observed in the leaf network but not in other plant parts while the connectors identified as *Bradyrhizobium* and *Burkholderia* were observed in the root network but not in the networks of the other plant parts.

## Discussion

At the Karoonda experimental site located in a Mediterranean climate, the three C4 perennial grasses investigated exhibited good growth in both the 2014 and 2015 summer seasons. The total plant biomass observed in this study was less than that previously reported for these grasses (Descheemaeker et al., 2014; Gupta et al., 2014) which was likely due to the seasonal variation in environmental factors, especially rainfall. There were significant differences in growth between the three grass species, especially in the 2014 season. In spite of the lower production of biomass, low soil mineral N level coupled with lower soil fertility and no fertilizer application necessitated the grass species to acquire the required N for growth from other sources, such as diazotrophic N fixation. Free-living diazotrophs can exhibit 10 times higher N fixation activity (25–50 mg N fixed g^−1^ protein h^−1^) than symbiotic N fixers (2–5 mg N fixed g^−1^ protein h^−1^) under ideal conditions (Mulder, 1975).

Previously, non-symbiotic (NS)-N fixation rates associated with the rhizosphere soil and roots of these three perennial grasses have been reported to range from 0.76 to 2.35 mg N kg^−1^ day^−1^, with N fixation rates higher in roots than in rhizosphere soil (Gupta et al., 2014). The range of N fixation potentials measured in this study are in the similar range of 0.5–4.0 mg N kg^−1^ day^−1^. In this study, we measured N fixation by diazotrophs residing within plant tissue only. Other reports of NS-N fixation, using the same method as the present study, for the warm season C4 grass *Panicum virgatum* showed a wider range of NS N fixation ranging from 0.003 to 4.6 mg N kg root^−1^ day^−1^ (Roley et al., 2018, 2019). While our measurements were done at one time point during peak growing season, the referenced study (Roley et al., 2019) included multiple points including a post-senescence time period. Previously, Gupta et al. (2014) reported lower levels of N fixation (0.05 to 0.27 mg N kg soil^−1^ day^−1^) in the rhizosphere soils collected during the post-senescence period. It should be noted that the amounts of NS-N fixation reported in this study are only an estimate of N_2_ fixation potential as they are measured in a laboratory assay under optimal conditions and could thus vary from *in-situ* field conditions. In addition, under field conditions N fixation could be episodic, depending up on the prevailing conditions. Roley et al. (2019) reported average NS-N fixation rates of 3.8 mg N kg root^−1^ day^−1^ for the North American perennial grass *Panicum virgatum* L. and further suggested that fixation may only be occurring episodically in response to transiently suitable environmental conditions and due to the presence of appropriate N-fixing bacterial populations. While we did not measure mycorrhizal colonization of these perennial grasses, arbuscular mycorrhizal fungi and N_2_-fixing bacteria have potentially complementary roles in providing the P and N needs of crops. However, Bauer et al. (2012) found no significant synergistic interactions between AMF and N_2_-fixers in prairie grassland communities and that any short-term effects of the two groups could only be additive. It can be assumed that as both these symbionts would compete for photosynthetic resources, any interactions between them may ultimately depend upon soil fertility, plant nutrient status and overall plant health (Mishra et al., 2008; Bauer et al., 2012).

### Relative Importance of NS-N Fixation to N Nutrition of Grasses

Due to an extensive root system and greater rhizosphere volume, some perennial grasses may promote microbial activity and improve N mineralisation (Ellis et al., 2008; Gupta et al., 2014). However, in the low organic matter sandy soils present at the experimental site, the contribution of N from soil organic matter mineralization was estimated to be <20 kg N ha^−1^ y^−1^ (Gupta et al., 2012; McBeath et al., 2015) and atmospheric N deposition in the remote/rural regions of southern Australia is estimated at <1 kg N ha^−1^ y^−1^ (Jeff Ladd, CSIRO, personal communication). Therefore, it is highly likely that NS-N fixation by rhizosphere and plant associated diazotrophs contribute a significant portion of the grass N requirement.

The significance of NS-N fixation for the N nutrition of sugarcane crops is well-established (Baptista et al., 2013). Similarly, recently Van Deynze et al. (2018) reported that atmospheric nitrogen fixation by mucilage-associated diazotrophic microbiota contributed 29–82% of the N nutrition of Sierra Mixe maize. Based on measurements of NS-N fixation potentials and coupled with mass balance estimates, Roley et al. (2018) suggested that NS-N fixation is an important source of N to un-fertilized temperate prairie grasses in North America. Overall, these observations are less than what is reported for sugarcane systems that indicate NS-N fixation rates of >40 kg N ha^−1^ y^−1^ (Urquiaga et al., 2012). The total percent contribution of BNF to total plant N for four C4 grasses (*Axonopus affinis, Paspalum notatum, Andropogon lateralis*, and *Aristida laevis*) was calculated to range from 22 to 36% of shoot total N (Marques et al., 2017), although the total input into the ecosystem varies due to differences in plant dry matter input. This resulted in estimated contributions of 0.6 to 37.6 kg N ha^−1^ among the four grasses. In the rain-fed cropping regions of Australia non-symbiotic/associative N fixation potentials were estimated to vary between 1 and 38 kg N ha^−1^ y^−1^ but could be agronomically significant when N fixation occurs during peak crop requirement such as during grain filling periods in crops (Gupta et al., 2006; Roper and Gupta, 2016).

### Coverage by Current Primers

**Figure 5:**
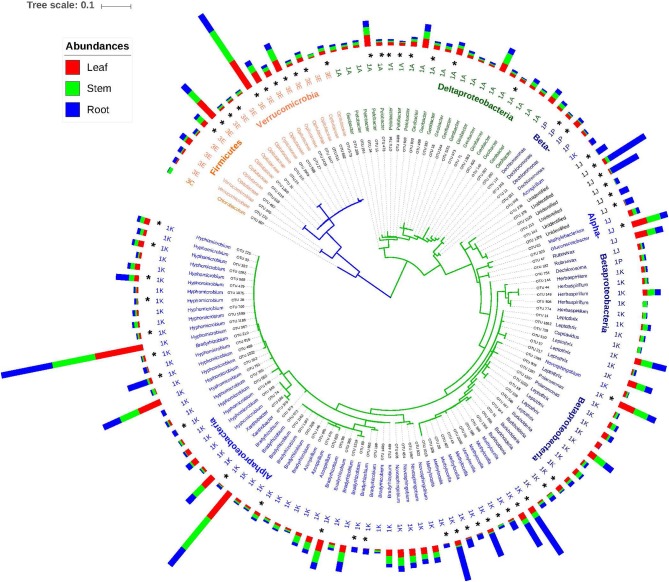
Neighbor joining tree based on top 134 OTUs (>0.1%) with taxonomic, nifH cluster and the mean abundance. The outer bars represent relative abundances for each OTU in the different plant part samples.

Based on the phylogenetic relationships between the *nifH* and 16S rRNA genes, four clusters of diazotrophs have been identified (Zehr et al., 2003; Raymond et al., 2004). Cluster I is generally identified as the most abundant in environmental samples and is comprised of aerobic and facultative anaerobic N fixers. An alternative nitrogenase *anfH*, a paralog of *nifH*, is included in cluster II. The deeply diverging cluster III (Figure 5) includes primarily obligate anaerobes, while cluster IV contains paralogs of the nitrogenase gene (Fujita et al., 1992; Raymond et al., 2004; Nomata et al., 2006; Staples et al., 2007).

The choice of *nifH* primers can influence on the recovery of *nifH* sequences due to the variation in their coverage. For example, although Zf/Zr primers indicate higher theoretical recovery of *nifH* diversity, the polF/polR primers to provide higher performance on the bench (Gaby and Buckley, 2017), though, based on bioinformatic analyses, they preferentially amplify Proteobacteria (Penton et al., 2013, 2016; Wang et al., 2013). Angel et al. (2018) recently evaluated primers targeting *nifH* using datasets created by mining metagenomic datasets and curated databases to create HMM models for filtering homologs. Their analysis of coverage revealed varying levels of bias among the tested primer sets (polF/polR were not included). These types of PCR biases have been documented across a number of *nifH* primers (Gaby and Buckley, 2012). However, the polF/polR primers used here have been verified to not significantly demonstrate quantifiable template-based bias, as compared to other *nifH* primers which exhibited up to a 1,000-fold mis-estimation of *nifH* gene copy numbers (Gaby and Buckley, 2017).

Given the fact that a primer set for protein coding genes cannot be comprehensive, our results of NifH sequence diversity measurements and community composition does not provide a complete picture of NifH diversity or a comprehensive representation of the community. In addition, there are limitations when assigning taxonomy to *nifH* sequences. Among these, the horizontal transfer (HGT) of the *nifH* gene (Zehr et al., 2003; Gaby and Buckley, 2012) imparts difficulties in resolving verifiable taxonomic relationships that are based on the 16S rRNA gene. While we chose to cluster at a 5% amino acid dissimilarity (Penton et al., 2016), NifH amino acid distances vary within different phylogenetic clusters, from 12.8% in the Alpha-Proteobacteria to 41.7% in the methanogens (Zehr et al., 2003). As such, a standard amino acid dissimilarity cut-off for OTU generation can result in the under- or over-estimation of diversity, depending on the bacterial group (Penton et al., 2016). Lastly, the deeper divergence of group III vs. group I (Figure 5) observed in our data is supported by earlier findings (Penton et al., 2016) and suggests that this taxonomic differentiation is more reliable, compared to the lower substitution rates within the much broader group 1 sequences (Gaby and Buckley, 2012).

### Diazotrophic Community

Non-leguminous plant species including cereals, grasses and other species such as maize, rice, sugarcane to harbor diazotrophic communities (Röesch et al., 2008; Prakamhang et al., 2009; Burbano et al., 2011; Sessitsch et al., 2012). Well-known examples of diazotrophic rhizosphere associations include *Azospirillum brasilense* and *Azospirillum lipoferum* within sugarcane (Hartmann et al., 2009). While diazotrophs in roots and the rhizosphere have received greater attention, their presence in the above ground plant parts such as the leaves and stems highlight their potential functional significance. Soils harbor an extensive diversity of bacteria. Accordingly, the microbial community composition and abundance within the plant rhizosphere and that of bacterial endophytes can vary according to soil type, management (including fertilizer application), season, plant type, plant growth stage and varieties (Bowen and Rovira, 1999; Donn et al., 2014; Gupta et al., 2014; Bouffaud et al., 2016). Overall, our findings show that the summer-active C4 perennial grasses, *Panicum, Digitaria* and Rhodes grass harbored diverse diazotrophic communities in both the above- and belowground parts of the plant. Significant differences in alpha-diversity were identified between the three grass species and between the different plant parts for each individual grass species (though there was no overall significance). Richness in the *Digitaria* grass was highest in leaves whereas the roots and stems harbored significantly higher richness in the *Panicum* and Rhodes grass, respectively. Previous reports with switchgrass and maize indicated greater diversity in the roots than in the shoots (Röesch et al., 2008; Bahulikar et al., 2014). However, Bahulikar et al. (2014) included both the rhizoplane and phyllosphere communities in their analysis whereas our results represent only genuine endophytic populations.

Alpha-Proteobacteria were the dominant group of *nifH*-harboring taxa both in the belowground (roots) and aboveground (leaves and stems) of the three perennial grass species. Dominance of Alpha-Proteobacteria is common, as they are widespread in terrestrial and aquatic environments (Wang et al., 2013; Collavino et al., 2014; Jing et al., 2015; Penton et al., 2016; Meng et al., 2019). Beta- and Delta-Proteobacteria were other dominant Proteobacterial groups. Similar observations were reported for switchgrass grown in Oklahoma and Michigan (Kellogg Biological Station, KBS) in the USA (Bahulikar et al., 2014; Roley et al., 2019). For example, there is a striking similarity in the most abundant endophytic *nifH* clusters and genera in the above ground plant parts observed in this study and that reported for the grass samples from Michigan but the roots of KBS samples were quite different even at phylum level (Figure S2). This difference is mainly due to the abundance to sequences related to *Dickeya* (*Dickeya dadantii*_Ech703, 63.7%) and *Clostridium* sp. in KBS-roots, which were almost absent in the Australian samples. The top seven Classes, Alpha-, Beta-, Delta-, and Gamma Proteobacteria, Opitutate, Verrucomicrobiae and Firmicutes, accounted for over 80% of all NifH sequences and were similar between the grasses in Australia and Switchgrass studies in the USA. Cluster IV sequences were not abundant in any of the Australian samples, although they have been shown to be associated with mangrove roots (Flores-Mireles et al., 2007).

Ultimately, endophytic colonization is principally through root infection by bacteria originating from the bulk soil that are influenced by root phenotypic properties. Higher diazotroph diversity in the rhizosphere than the roots among different grass species suggests a plant-based selection from the rhizosphere microbial community (Gupta et al., 2014). This lower total endophytic bacterial diversity in the leaves and stems, compared to the roots, may also be due to the exposure to stressors such as desiccation, reactive oxygen, UV radiation, and lack of nutrients (Lindow and Brandl, 2003; Ma et al., 2013). In other cases, diazotroph richness was lower in roots, perhaps the metabolically rich environment of the roots supports a broader niche diversity than that present in the N-poor stem and leaf environments (Videira et al., 2012). Plant tissue type more strongly dictated the composition of the endophytic diazotrophic community in rice over the type of soil or fertilizer amendment (Prakamhang et al., 2009). However, it appears that this influence of tissue type on endophyte diversity is plant dependent. For example, extensive taxonomic overlap was observed between the leaf and root microbiota in Arabidopsis (Bai et al., 2015). Endophytic compartments of different *M. giganteus* plants have been reported to harbor similar microbial communities across all sites, whereas the rhizosphere soil of different plants tended to harbor diverse microbial assemblages that were distinct among sites (Lundberg et al., 2012; Li et al., 2016). However, presence alone may not necessarily reflect activity within the plant tissue. For example, DNA-based analyses of diazotrophs in elephant grass indicated that plant tissue type was the strongest driver of community composition. However, mRNA based analyses contradicted this finding, with plant genotype driving diazotroph community composition (Videira et al., 2013). Abundances of *nifH* gene harboring bacteria ranged from 8.4 × 10^5^ to 1.2 × 10^7^ g^−1^ plant material, with the highest abundances in the roots, followed by the stems and leaves. Previously, the abundance of diazotrophic populations was determined to range between 10^3^ and 10^7^ g^−1^ plant material in elephant grass roots and stems (Reis et al., 2001; Videira et al., 2012) and 10^6^ copies in the rhizosphere and roots of several perennial grasses (Gupta et al., 2014). Overall, there was no consistent correlation between copy numbers and diversity indices. However, there appears to be an inverse relationship within the roots. *Digitaria* roots contained the highest *nifH* copy number but lowest diversity. In contrast, *Panicum* roots harbored the lowest abundance with higher diversity. These results support that plant-specific factors that drive root phenotype influence both the diversity and abundance of endophytic diazotrophs. Overall, these results confirm previous reports of plant species-based variation in diazotrophic community composition, albeit these results are first reports of diazotroph community diversity among perennial grasses grown under the same environment. Furthermore, these results confirm the complexity of the endophyte diazotroph community, indicating that selection pressures that impact endophyte diversity are plant species dependent and are further impacted by tissue type in these grasses.

### Individual Dominant Genera

Despite identifying a broad diversity of *nifH*-harboring taxa, the endophytic community was dominated by only a few genera (17) within seven subcluster groups. Bacterial genera such as *Hyphomicrobium, Bradyrhizobium, Geobacter, Azospirillum, Diplosphaera, Burkholderia*, and *Methylocella* were dominant in all the three grass species and across plant parts, which is in agreement with previous studies (Bahulikar et al., 2014; Roley et al., 2019). Direct comparisons to these studies indicate a strong similarity in the most abundant genera in the aboveground biomass, though there is a striking dissimilarity within the roots (Figure S2). These dominant genera contributed most to the differences among plant parts and grass types. For many of these genera, little is known concerning their ecological role, especially in terms of N fixation. However, they are commonly recovered in soil and rhizosphere 16S rRNA gene and *nifH*-based culture-independent studies. *Hyphomicrobium* has been identified in a wide variety of environments (Oren and Xu, 2014), as a diazotroph in sugarcane (Dong et al., 2018) and as endophytes within taro (Nayak et al., 2016) and rice (Mano and Morisaki, 2008), dominated the rhizosphere of eucalyptus (da Silva et al., 2015), and is part of the core microbiome in panicles of the weeds *Setaria pumila* and *Setaria viridis* (Rodriguez et al., 2018). The genus *Burkholderia* contains several confirmed diazotrophic species such as *Burkholderia vietnamiensis* (Gillis et al., 1995), *B. kururiensis* (Zhang et al., 2000), and *B. brasilensis* (Hartmann et al., 1995) who penetrates root cells through damaged membranes (Baldani et al., 1995) and secondary root points of emergence (Baldani et al., 1986), and colonizes the stomata of rice seedlings (Silva et al., 2000). Identified in grass and cereal rhizospheres worldwide, the genus *Azospirillum* contains members widely known as plant growth promoting rhizobacteria and free-living N-fixers within the rhizosphere and as endophytes (see Steenhoudt and Vanderleyden, 2000 for a comprehensive review). Significant populations (10^5^ to 10^6^) of *Azospirillum* were recovered in the endorhizoplane, rhizoplane, and rhizosphere of C4 grasses (Marques et al., 2017). *Methylocella* is a methanotroph that has been identified, through *nifH* gene sequencing, to be the most prevalent taxa in cassava, maize, and sugarcane (Reinhardt et al., 2008), though it was largely restricted to the soil rather than the roots and stems of maize in another study (Röesch et al., 2008). *Geobacter* has been found as an endophyte of moss (Liu et al., 2014), rice (Sun et al., 2008), and several grasses (Hamelin et al., 2002; Bahulikar et al., 2014; Wemheuer et al., 2017). *Bradyrhizobium*, is an acid-tolerant, slow-growing rhizobia (Graham, 1992; Koponen et al., 2003) capable of both N_2_ fixation (Hara et al., 2019), and denitrification (Bedmar et al., 2005; Sánchez et al., 2011) and is among the most abundant in *nifH* sequence (Thaweenut et al., 2011; Meng et al., 2019) and shotgun metagenomic based studies (Hara et al., 2019). most abundant N fixer associated with sorghum roots (Hara et al., 2019) and the stems and tubers of African sweet potatoes (Reiter et al., 2003). Bradyrhizobia and *Azorhizobium* were also identified as the most abundant endophytes of sugarcane stems and roots (Thaweenut et al., 2011) with *Bradyrhizobia* also found most abundant in switchgrass (Roley et al., 2019). Lastly, among the most prevalent *nifH*-harboring bacteria identified, *Diplosphaera* is represented by an N-fixing isolate from a termite hindgut (Wertz et al., 2011). This reference name has been corrected to *Geminisphaera colitermitum* [(Wertz et al., 2011) (second correction)]. Our data indicates that this endophyte is also closely related to Opitutaceae bacterium TAV2, thus the classification to the original *Diplosphaera* is likely problematic. TAV2 has been identified as a prevalent *nifH* harboring endophyte of *Oxyria digyna* and *Saxifraga oppositifolia* in an alpine system (Kumar et al., 2017) and prevalent in anaerobic permafrost soils (Penton et al., 2016).

### Diazotroph Co-occurrence Networks

**Figure 6:**
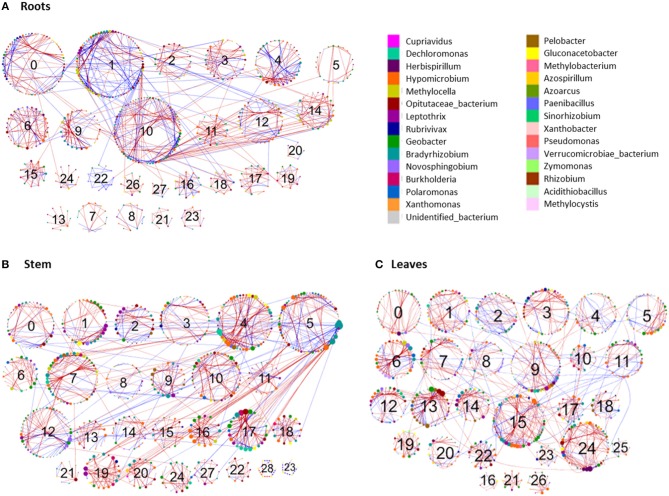
Comparison of diazotroph community networks associated with different plant parts of the three perennial grass species. Circles represent nodes whose size indicates connectivity; node color represents taxonomy at the genus level. Edges indicate co-occurrence between nodes colored either blue for positive or red for negative. Each circular grouping is a module, modules containing at least five nodes are identified by their assigned number.

The individual plant part networks were larger and more complex, with more nodes, links, and particularly, modules, than the whole-plant network (Figure 6, Figure S4). This suggests that co-occurrence patterns within the *nifH-*harboring community are compartmentalized to different parts of the plant, that is, that each part of the plant represents a unique niche or microsite whose members interact to a larger degree with members within that niche. Indeed, previous results have shown endophyte networks with bacterial terminal restriction fragments (T-RF) in *Phragmites australis* (Ma et al., 2013). The finding that the majority of overall links were negative indicates that competition for resources (e.g., C), space, or other antagonistic properties were more prevalent than cooperation or mutualism. A limited number of suitable niches within the endophytic environment may also contribute to the more negative interactions. This is in comparison to previous studies that identified a greater number of positive links, such as within the rhizosphere (82–94% positive) (Shi et al., 2016). Negative interactions in the root, stem, and leaf networks were more abundant within a module, with a higher proportion of positive links occurring between modules. This suggests that modules which are considered as individual functional units in a network may represent individual microsites with commonality in niche requirements (Shi et al., 2016), with competition or antagonism occurring within. Conversely, the lesser number of positive co-occurrences between modules indicates facilitative or mutualistic interactions, though there is evidence that classically negative antagonistic interactions can be included in positive interactions (Toju et al., 2016). The number of nodes, links, modularity, and harmonic geodesic distance all increased from the roots to the stem to the leaves. As these endophytic bacteria primarily originate from the soil, colonize root tissue, and migrate through the stem to the leaves, this may illustrate a selective maturation of the *nifH*-harboring community over time and space. Notably, there are other sources of endophyte colonization, such as within the spermosphere, anthosphere, caulosphere and phyllosphere (Mano and Morisaki, 2008; Compant et al., 2012) and colonization through these paths could not be discounted. In this study, there does not appear to be a strong selection during movement to the leaves, as illustrated by non-significant changes in alpha-diversity. However, the higher number of shared OTUs (Figure 2A) between the root-stem and stem-leaf does suggest some low-level selective events.

Module hubs are strongly interconnected species that may be important for plant health and mediate interactions between the plant and the endophytic community (Agler et al., 2016). Microbial hubs may indirectly influence other taxa by altering the quality or performance of the plant host without direct interaction with other taxa (van der Heijden and Hartmann, 2016). Module hubs were most prevalent in the stem and leaves, with few identified in the roots. Differences in the number and identity of module hubs and connectors among the different plant parts also supports the perspective that the differences in the local niche environments within each plant part are allowing for the proliferation of specific community members and different interactions. In addition, host plant defense mechanisms may also select against different bacteria, and these defenses may vary temporally. These types of interactions were observed with the inoculation of rice with *Herbaspirilllum* cells, with differences observed even between cultivars (James et al., 2002).

There were three unique module hubs identified in the leaf network represented by *Herbaspirillum, Leptothrix*, and *Methylobacterium*. Of these, *Herbaspirillum* is the most widely recognized plant growth promoting bacteria (see Monteiro et al., 2012 for a comprehensive review of this genus). They are known to colonize the root system first, move into the xylem, spread into the stem and leaves, and finally spread into substomal cavities and intercellular spaces (Monteiro et al., 2012). Another leaf module hub was identified as *Leptothrix*, a genus previously isolated from roots of the common bean (*Phaseolus vulgaris*) (López-López et al., 2010) and cattail (*Typha angustifolia*) (Li et al., 2011), and identified as the most common diazotroph in elephant grass stems (Videira et al., 2013). The last leaf network hub was *Methylobacterium*, a methylotroph that is capable of utilizing methanol emitted from the stomata of some plants (Nemecek-Marshall et al., 1995). They are reported to be one of the most abundant genera in the phyllosphere and colonize plants as endophytes, epiphytes, and some tissues intracellularly (Corpe and Rheem, 1989; Hirano and Upper, 1991; Mcinroy and Kloepper, 1995; Elbeltagy et al., 2000; Pirttila et al., 2000; Delmotte et al., 2009). Due to their ability to use various C sources and their coevolution with plants, these bacteria may have evolved into generalists, rather than specialists, leading to their high occurrence within and on leaves (Knief et al., 2010).

### Stochastic vs. Deterministic

Haruna et al. (2018) suggested that stochastic processes dominate in the community assembly for bacterial endophytes in rainforest plant species as bacterial endophyte OTUs were randomly distributed among plant organs and rhizosphere soil indicating that different plant parts may be offering similar environments. Our results for a specific functional group indicate that diazotrophic (*nifH* harboring) communities in all plant parts contain OTUs that are spread randomly across the phylogenetic tree at Phylum/Class level (NRI <0) but are also phylogenetically clustered toward the tips (NTI > 0). Thus, due to neutral selection at coarser phylogenetic levels, that this putatively beneficial functional gene (*nifH*) is not the key determinant for selection or colonization in aboveground plant parts. Rather, deterministic selection is relegated to closely related OTUs, suggesting that individual spatial compartments within the plant may select according to the fitness of an individual due to the resources and interactions that occur within that niche. This selection may be due to factors such as the expression of constitutive genes (e.g., stress response) or metabolic attributes such as the utilization of specific electron acceptors that allow for survival within a specific plant spatial compartment (niche). In addition, differences in fitness among closely related taxa may be related to survival within the context of the plant host defense responses, able to pass over several barriers while colonizing aboveground plant parts and possess the physiological requirements to establish in different plant niches (Compant et al., 2010).

### System Level Perspective

Agricultural soils in the Mediterranean climatic region of southern Australia are generally low in soil organic matter (organic C <1%) and N supply capacity (McBeath et al., 2015). For example, (Descheemaeker et al., 2014) observed <20 kg of mineral N in the soil profile up to 1 M depth in this field experiment. Additionally, the summer active perennial grasses investigated in this study didn't receive any external fertilizer application, hence they are solely dependent on the soil N and thus N input from diazotrophic N fixation becomes a significant source of plant N requirement (25–50 kg per ha). The biomass production for the perennial grasses depends up on summer rainfall and the grasses were in active growth with high N requirements during experimental sampling period of this study (Descheemaeker et al., 2014). The deep and extensive root system of the perennial grasses in the sandy-textured soils at the experimental location provide a C-rich environment for diazotrophic N fixation through their dense rhizosphere environment with significant inputs of C through rhizodeposition. However, diazotrophs in the rhizosphere are exposed to dry and hot conditions therefore conditions for N fixation, i.e., in terms of soil moisture and anaerobic/microaerophilic conditions, would only occur episodically (~10 during the summer period). Gupta et al. (2014) observed potential rates of 0.84–1.4 mg N fixed kg^−1^ soil day^−1^ for the rhizosphere soils of these grasses. Unlike the rhizosphere communities, the endophytes would not be exposed to extreme weather conditions, especially in terms of moisture status, hence potentially can make a significant contribution to plant N requirements. However, as the diazotrophs depend on plants for the C inputs as an energy source of N fixation, conditions external to plant would influence the diazotroph-plant interactions and N fixation. The episodic nature of diazotrophic N fixation makes it difficult to extend the short-term measurements of N fixation reported in this study to the entire crop season (Roley et al., 2018). Overall, the presence of diazotrophs in all plant parts with potential for N fixation would provide agronomically significant N inputs to meet crop N requirements in particular during rapid growth and biomass production of the unfertilized grasses. Similarly, more than 45% of crop N for cereal crops has been found to be contributed by sources other than fertilizer N and it is suggested that NS-N_2_ fixation may be contributing a major portion of this N requirement (Ladha et al., 2016; Roper and Gupta, 2016).

## Conclusions

Summer-growing perennial grasses investigated in this study, i.e., *Panicum coloratum* L. cv. *Bambatsi* (Bambatsi panic), *Chloris gayana Kunth* cv. *Katambora* (Rhodes grass), and *Digitaria eriantha* Steud. cv. Premier (Premier digit grass) growing in the poor fertility sandy soils in the Mediterranean regions of southern Australia and Western Australia solely depend upon soil N and biological N inputs through diazotrophic (free living or associative) N fixation. These grasses found to support a diverse and abundant diazotrophic community in the rhizosphere soil and inside plant both above and below-ground. The three grasses investigated in this study have shown N fixation potentials ranging between 0.5 and 4.0 mg N/kg/day and N fixation capacity found in both the above (leaves) and below ground (roots) plant parts. In general, there was a limited difference in the diversity between leaves, stems and shoots except that Panicum grass roots harbored greater species richness. Results also confirmed previous reports of plant species-based variation in diazotrophic community and Alpha-Proteobacteria were the dominant group of *nifH*-harboring taxa both in the belowground (roots) and aboveground (leaves and stems) of the three perennial grass species. Results also show a well-structured *nifH* community in all plant parts, first report of this type for a functional community particularly endophytic community. The presence of distinct endophytic diazotrophic assemblages and the variation in the number and identity of module hubs and connectors among the different plant parts suggesting that local environments of the niches within each plant part may dictate the overall composition of diazotrophs within a plant.

## Data Availability Statement

The datasets for the *nifH* sequences and associated metadata generated for this study can be found at NCBI-SRA with the bioproject ID: PRJNA550285.

## Author Contributions

VG and JT contributed to all aspects of the study. BZ conducted the sequencing. BZ, CP, and JY contributed to bioinformatics and network analysis. All the authors contributed to the preparation of the manuscript.

### Conflict of Interest

The authors declare that the research was conducted in the absence of any commercial or financial relationships that could be construed as a potential conflict of interest.
