# Supplementary material for: Diazotroph Diversity and Nitrogen Fixation in Summer Active Perennial Grasses in a Mediterranean Region Agricultural Soil
Source: Front Mol Biosci. 2019 Nov 5;6:115. doi: 10.3389/fmolb.2019.00115 (PMC6848460; doi:10.3389/fmolb.2019.00115)

Table S1. Physico-chemical properties of soils, plant biomass and site weather from the experimental plots with different summer-active perennial grasses at Karoonda, South Australia during the 2014 and 2015 plant growth period.

Property	Digitaria	Panicum	Rhodes grass
pH-water	7.11	6.94	6.93
Organic C (%)	0.89 (0.08)	0.70 (0.04)	0.90 (0.08)
Total N (%)	0.05 (0.007)	0.03 (0.005)	0.05 (0.007)
C:N ratio	16.5 (0.7)	20.4 (1.5)	16.7 (0.5)
Dissolved Org C (ug C/g)	57.2 (0.7)	50.6 (2.3)	64.7 (8.2)
Mineral N (ug N/g)	3.8 (0.08)	2.4 (0.0)	2.5 (0.3)
Sand (%)	74.7 (0.9)	76.2 (1.9)	76 (0.7)
Clay (%)	6.1 (0.6)	6.9 (0.6)	6.8 (0.4)
CEC (cmol(+)/kg	3.6 (0.3)	3.5 (0.4)	3.5 (0.4)
Plant Biomass (t/ha)			
Dec-13	0.116 (0.049)	0.129 (0.028)	0.183 (0.022)
Apr-14	1.045 (0.318)	1.447 (0.358)	0.682 (0.217)
Jan-15	0.248 (0.036)	0.705 (0.101)	0.683 (0.063)
Apr-15	0.356 (0.088)	0.863 (0.088)	0.713 (0.104)
Rainfall (mm)			
Jan-April 2014		151	
Jan-April 2015		121	
Max Temp (oC)		29.1 (4.6)	
Min Temp (oC)		12.5 (2.4)	

Table S2. Additional topological properties of molecular ecological networks for nifH gene harboring bacterial communities.

Network Indexes	Roots	Leaf	Stem	All parts
Average path distance (GD)	6.969	9.788	8.123	4.992
Geodesic efficiency (E)	0.174	0.127	0.15	0.237
Harmonic geodesic distance (HD)	5.742	7.9	6.684	4.226
Maximal degree	18	17	23	27
Nodes with max degree	OTU_2127; OTU_2924	OTU_2509	OTU_0096	OTU_0293
Centralization of degree (CD)	0.021	0.017	0.026	0.069
Maximal betweenness	20592.558	37135.673	64591.116	7924.244
Nodes with max betweenness	OTU_2831	OTU_0125	OTU_0096	OTU_0293
Centralization of betweenness (CB)	0.098	0.12	0.24	0.127
Maximal stress centrality	573641	4054851	2964658	80019
Nodes with max stress centrality	OTU_0272	OTU_4485	OTU_1729	OTU_0293
Centralization of stress centrality (CS)	2.673	13.291	11.02	1.296
Maximal eigenvector centrality	0.285	0.306	0.312	0.374
Nodes with max eigenvector centrality	OTU_2127;OTU_2924	OTU_0695;OTU_3180;OTU_2512	OTU_0095;OTU_1924	OTU_1090
Centralization of eigenvector centrality (CE)	0.276	0.3	0.304	0.349
Density (D)	0.007	0.006	0.006	0.009
Reciprocity	1	1	1	1
Transitivity (Trans)	0.203	0.175	0.137	0.083
Connectedness (Con)	0.688	0.792	0.826	0.655
Efficiency	0.992	0.995	0.994	0.99
Hierarchy	0	0	0	0
Lubness	1	1	1	1

Table S3. Topological properties of the empirical molecular ecological networks (MENs) of each plant part and their associated random MENs.

Empirical Network Indexes						100 Random Networks Indexes		
Plant part	Similarity Threshold	R^2 of Power Law	Average clustering coefficient (avgCC)	Average Path (GD)	Modularity (Fast greedy)	Average clustering coefficient (avgCC)	Average Path (GD)	Modularity (Fast greedy)
Roots	0.92	0.742	0.156	6.969	0.846	0.010 ± 0.002	4.192 ± 0.025	0.461 ± 0.004
Stem	0.91	0.729	0.141	8.123	0.872	0.008 ± 0.002	4.412 ± 0.019	0.482 ± 0.005
Leaf	0.91	0.744	0.141	9.788	0.908	0.007 ± 0.002	4.545 ± 0.023	0.498 ± 0.005
All	0.72	0.908	0.097	4.992	0.685	0.018 ± 0.006	4.300 ± 0.068	0.577 ± 0.006

Table S4. Node topology of molecular ecological networks for NifH harboring bacterial communities.

Node Classification	Plant part	OTU	BLAST - Genus
Network Hub	All	0293	Unidentified_bacterium_nif_cluster
Module Hub	All	1090	Unidentified_bacterium_nif_cluster
		0214	Burkholderia_sp_Ch1-1_ctg00023
		1353	Geobacter_uraniireducens_Rf4
		0502	Methylocella_silvestris_BL2
		0922	Opitutaceae_bacterium_TAV2_ctg796
		1751	Rubrivivax_benzoatilyticus_JA297
		0909	Leptothrix_cholodnii_SP-6
	Roots	0772	Hyphomicrobium_sp_MC1
		1039	Hyphomicrobium_sp_MC1
		3020	Hyphomicrobium_sp_MC1
	Stem	0096	Bradyrhizobium_sp_ORS278
		3587	Geobacter_uraniireducens_Rf4
		5277	Geobacter_uraniireducens_Rf4
		0082	Hyphomicrobium_sp_MC1
		2837	Hyphomicrobium_sp_MC1
		0557	Hyphomicrobium_sp_MC1
		2508	Hyphomicrobium_sp_MC1
		0361	Novosphingobium_nitrogenifigens_DSM_19370_00023
		0492	Novosphingobium_nitrogenifigens_DSM_19370_00023
		2153	Opitutaceae_bacterium_TAV2_ctg796
		0843	Rubrivivax_benzoatilyticus_JA297
	Leaf	3619	H_seropedicae_nifD
		0767	Geobacter_uraniireducens_Rf4
		0320	Methylobacterium_nodulans_ORS_2060
		2115	Bradyrhizobium_sp_ORS278
		3972	Leptothrix_cholodnii_SP-6
		1989	Leptothrix_cholodnii_SP-6
		0256	Geobacter_sp_FRC-32
Connectors	All	1860	Gluconacetobacter_diazotrophicus_PAl_5_complete_genome_
		2143	Bradyrhizobium_sp_ORS278
		1561	Leptothrix_cholodnii_SP-6
		1154	Geobacter_uraniireducens_Rf4
		3587	Geobacter_uraniireducens_Rf4
		1615	Leptothrix_cholodnii_SP-6
		0457	Methylocella_silvestris_BL2
	Roots	1862	Leptothrix_cholodnii_SP-6
		0163	Burkholderia_sp_Ch1-1_ctg00023
		2546	Unidentified_bacterium_nif_cluster
		1490	Bradyrhizobium_elkaniifor_dinitrogen_reductase
	Stem	4184	Gluconacetobacter_diazotrophicus_PAl_5_complete_genome_
	Leaf	0125	Hyphomicrobium_sp_MC1
		0520	Leptothrix_cholodnii_SP-6
		1635	Hyphomicrobium_sp_MC1
		0301	Geobacter_sp_FRC-32

Table S5. Phylotypes and closest relatives of NifH sequences associated with different plant parts of summer-active perennial grasses.

Phylotype	Closest relative	Accession no.	Frambot			Relative abundance (%)				Taxonomic description
			Similarity (%)	Score	Framshifts	Leaf	Steam	Root	Mean	
OTU_367	Hyphomicrobium sp. MC1	CCB66897	98	522	0	3.93	3.52	4.19	3.88	Alphaproteobacteria
OTU_672	Bradyrhizobium sp. ORS278	CAL79071	96	514	0	2.69	3.20	1.73	2.54	Alphaproteobacteria
OTU_2909	Opitutaceae bacterium TAV2	EEG22235	93	474	2	2.61	2.90	1.61	2.37	Verrucomicrobia
OTU_562	Hyphomicrobium sp. MC1	CCB66897	95	461	3	1.93	1.62	1.49	1.68	Alphaproteobacteria
OTU_388	Methylocella silvestris BL2	ACK52517	96	500	1	0.51	0.45	3.55	1.50	Alphaproteobacteria
OTU_378	Burkholderia (endosymbiotic)	AAK26105	99	511	1	0.26	0.26	3.44	1.32	Betaproteobacteria
OTU_1260	Opitutaceae bacterium TAV2	EEG22235	95	466	3	1.76	1.05	0.76	1.19	Verrucomicrobia
OTU_328	Methylocella silvestris BL2	ACK52517	94	462	3	0.39	0.13	2.79	1.10	Alphaproteobacteria
OTU_909	Polaromonas naphthalenivorans CJ2	ABM37652	99	509	1	1.34	1.19	0.67	1.07	Betaproteobacteria
OTU_586	Methylocella silvestris BL2	ACK52517	96	502	1	0.55	0.41	2.09	1.02	Alphaproteobacteria
OTU_323	Gluconacetobacter diazotrophicus PAI 5	CAP54379	95	502	1	1.25	1.13	0.57	0.98	Alphaproteobacteria
OTU_370	Xanthobacter autotrophicus Py2	ABS65347	96	502	1	1.12	1.01	0.76	0.96	Alphaproteobacteria
OTU_1525	Burkholderia (endosymbiotic)	AAK26105	99	513	1	0.11	0.07	2.45	0.88	Betaproteobacteria
OTU_520	Leptothrix cholodnii SP-6	ACB33698	99	510	1	0.54	0.93	1.12	0.86	Betaproteobacteria
OTU_365	Bradyrhizobium sp. ORS278	CAL79071	99	505	1	0.93	0.96	0.53	0.80	Alphaproteobacteria
OTU_2435	Opitutaceae bacterium TAV2	EEG22235	97	516	1	0.93	1.00	0.47	0.80	Verrucomicrobia
OTU_359	Polaromonas naphthalenivorans CJ2	ABM37652	96	499	1	0.64	0.94	0.77	0.78	Betaproteobacteria
OTU_49	Methylocella silvestris BL2	ACK52517	96	494	3	0.27	0.10	1.96	0.78	Alphaproteobacteria
OTU_47	Rubrivivax benzoatilyticus JA297	EGJ10941	96	521	0	1.01	0.68	0.59	0.76	Betaproteobacteria
OTU_59	Bradyrhizobium sp. ORS278	CAL79071	98	504	1	0.58	0.79	0.78	0.71	Alphaproteobacteria
OTU_11	Pelobacter propionicus DSM 2379	ABL01060	95	498	1	0.92	0.65	0.48	0.68	Deltaproteobacteria
OTU_498	Hyphomicrobium sp. MC1	CCB66897	97	479	2	0.23	0.16	1.65	0.68	Alphaproteobacteria
OTU_217	Leptothrix cholodnii SP-6	ACB33698	96	500	1	0.74	0.74	0.55	0.68	Betaproteobacteria
OTU_71	Geobacter uraniireducens Rf4	ABQ25379	99	551	0	0.67	0.89	0.43	0.66	Deltaproteobacteria
OTU_508	Opitutaceae bacterium TAV2	EEG22235	94	441	4	0.91	0.60	0.40	0.64	Verrucomicrobia
OTU_830	Geobacter uraniireducens Rf4	ABQ25379	95	523	0	0.74	0.60	0.56	0.63	Deltaproteobacteria
OTU_429	Hyphomicrobium sp. MC1	CCB66897	97	479	2	0.37	0.43	1.04	0.61	Alphaproteobacteria
OTU_608	Novosphingobium nitrogenifigens DSM 19370	EGD60366	96	521	0	0.64	0.69	0.46	0.60	Alphaproteobacteria
OTU_454	Novosphingobium nitrogenifigens DSM 19370	EGD60366	94	487	1	0.57	0.56	0.41	0.51	Alphaproteobacteria
OTU_149	Herbaspirillum seropedicae SmR1	CAA90932	94	511	0	0.37	0.61	0.45	0.48	Gammaproteobacteria
OTU_449	Rhizobium sp. ORS571	AAA26316	96	492	1	0.50	0.65	0.26	0.47	Alphaproteobacteria
OTU_96	Bradyrhizobium sp. ORS278	CAL79071	98	482	2	0.48	0.60	0.30	0.46	Alphaproteobacteria
OTU_923	Novosphingobium nitrogenifigens DSM 19370	EGD60366	94	481	2	0.52	0.45	0.40	0.46	Alphaproteobacteria
OTU_1467	Novosphingobium nitrogenifigens DSM	EGD60366	94	483	2	0.54	0.40	0.37	0.44	Alphaproteobacteria
OTU_290	Geobacter bemidjiensis Bem	ACH39087	97	538	0	0.47	0.43	0.33	0.41	Deltaproteobacteria
OTU_1118	Opitutaceae bacterium TAV2	EEG22235	95	456	4	0.40	0.48	0.23	0.37	Verrucomicrobia
OTU_623	Azospirillum amazonense Y2 01430	EGX99768	99	527	0	0.27	0.59	0.22	0.36	Alphaproteobacteria
OTU_74	Hyphomicrobium sp. MC1	CCB66897	97	509	0	0.49	0.16	0.39	0.35	Alphaproteobacteria
OTU_16	Geobacter uraniireducens Rf4	ABQ25379	95	505	1	0.53	0.24	0.24	0.34	Deltaproteobacteria
OTU_487	Opitutaceae bacterium TAV2	EEG22235	90	425	3	0.47	0.18	0.37	0.34	Verrucomicrobia
OTU_125	Hyphomicrobium sp. MC1	CCB66897	97	481	2	0.45	0.34	0.21	0.33	Alphaproteobacteria
OTU_568	Hyphomicrobium sp. MC1	CCB66897	97	483	2	0.41	0.36	0.22	0.33	Alphaproteobacteria
OTU_27	Opitutaceae bacterium TAV2	EEG22235	96	491	2	0.47	0.31	0.19	0.32	Verrucomicrobia
OTU_332	Hyphomicrobium sp. MC1	CCB66897	96	496	3	0.46	0.30	0.18	0.31	Alphaproteobacteria
OTU_1022	Polaromonas naphthalenivorans CJ2	ABM37652	98	456	2	0.47	0.18	0.28	0.31	Betaproteobacteria
OTU_63	Methylobacterium nodulans ORS 2060	ACL58883	96	482	2	0.36	0.32	0.23	0.30	Alphaproteobacteria
OTU_99	Methylocella silvestris BL2	ACK52517	95	475	2	0.14	0.08	0.68	0.30	Alphaproteobacteria
OTU_238	Burkholderia (endosymbiotic)	AAK26105	93	477	1	0.01	0.04	0.85	0.30	Betaproteobacteria
OTU_140	Geobacter bemidjiensis Bem	ACH39087	98	521	2	0.23	0.33	0.28	0.28	Deltaproteobacteria
OTU_437	Burkholderia sp. Ch1-1	EFG73464	100	535	0	0.34	0.37	0.13	0.28	Betaproteobacteria
OTU_29	Methylocella silvestris BL2	ACK52517	94	452	4	0.10	0.04	0.68	0.27	Alphaproteobacteria
OTU_1357	Polaromonas naphthalenivorans CJ2	ABM37652	99	508	1	0.27	0.37	0.18	0.27	Betaproteobacteria
OTU_511	Opitutaceae bacterium TAV2	EEG22235	96	491	2	0.33	0.33	0.15	0.27	Verrucomicrobia
OTU_14	Leptothrix cholodnii SP-6	ACB33698	99	510	1	0.14	0.41	0.22	0.26	Betaproteobacteria
OTU_112	Dechloromonas aromatica RCB	AAZ46164	98	536	0	0.23	0.33	0.20	0.25	Betaproteobacteria
OTU_503	Pelobacter propionicus DSM 2379	ABL01060	95	488	2	0.39	0.22	0.14	0.25	Deltaproteobacteria
OTU_30	Hyphomicrobium sp. MC1	CCB66897	96	515	0	0.25	0.27	0.23	0.25	Alphaproteobacteria
OTU_1376	Burkholderia (endosymbiotic)	AAK26105	98	499	2	0.06	0.04	0.64	0.25	Betaproteobacteria
OTU_142	Burkholderia (endosymbiotic)	AAK26105	99	529	0	0.02	0.00	0.71	0.24	Betaproteobacteria
OTU_644	Burkholderia sp. Ch1-1	EFG73464	98	505	1	0.26	0.20	0.27	0.24	Betaproteobacteria
OTU_12	Dechloromonas aromatica RCB	AAZ46164	94	504	1	0.45	0.09	0.19	0.24	Betaproteobacteria
OTU_573	Opitutaceae bacterium TAV2	EEG22235	95	480	2	0.28	0.27	0.17	0.24	Verrucomicrobia
OTU_329	Bradyrhizobium sp. ORS278	CAL79071	99	507	1	0.26	0.30	0.15	0.24	Alphaproteobacteria
OTU_337	Geobacter uraniireducens Rf4	ABQ25379	94	478	2	0.21	0.24	0.22	0.23	Deltaproteobacteria
OTU_458	Geobacter uraniireducens Rf4	ABQ25379	94	474	2	0.34	0.19	0.11	0.21	Deltaproteobacteria
OTU_698	Burkholderia sp. Ch1-1	EFG73464	98	519	1	0.07	0.26	0.30	0.21	Betaproteobacteria
OTU_661	Azospirillum amazonense Y2 01430	EGX99768	99	505	1	0.18	0.31	0.11	0.20	Alphaproteobacteria

Table S6. Niche values[&] for plant-C and plant-N as indicators of abundance-weighted mean of the environmental variables for each *nif*H-OTU in the community and the significance is based on a p<0.05 (two-tailed test).

**Plant C niche values**					
Observed	Mean.Simulated	LowCI	UppCI	Significance at P<0.05	
OTU_2909	293.73	281.99	269.10	296.15	Non-sig
OTU_1017	299.45	282.16	261.46	302.94	Non-sig
OTU_2435	298.08	282.04	269.18	295.88	Higher
OTU_1118	296.46	282.20	263.50	300.67	Non-sig
OTU_1260	300.82	281.74	265.36	298.47	Higher
OTU_1189	310.05	281.25	243.56	314.21	Non-sig
OTU_1353	284.51	280.67	194.04	355.49	Non-sig
OTU_1151	287.29	282.27	236.90	324.82	Non-sig
OTU_1525	162.64	280.58	219.62	338.41	Lower
OTU_1376	185.69	280.35	212.93	337.23	Lower
OTU_1035	343.07	281.79	110.77	394.46	Non-sig
OTU_1308	223.86	282.17	207.59	351.14	Non-sig
OTU_1015	191.96	280.75	221.14	337.39	Lower
OTU_2080	236.38	281.00	225.52	330.93	Non-sig
OTU_1022	274.67	282.26	227.03	338.05	Non-sig
OTU_1367	291.94	281.63	247.90	313.99	Non-sig
OTU_1357	294.65	281.71	262.83	300.45	Non-sig
OTU_1862	293.18	281.17	225.05	327.32	Non-sig
OTU_1467	289.92	281.84	261.12	303.12	Non-sig
OTU_1219	284.98	280.83	250.08	312.36	Non-sig
OTU_1347	297.14	281.44	259.34	305.26	Non-sig
OTU_1162	298.73	281.28	238.04	323.12	Non-sig
OTU_1490	288.94	281.46	242.12	320.48	Non-sig
OTU_1032	277.15	280.87	227.13	325.58	Non-sig
OTU_1975	277.48	280.88	253.96	309.02	Non-sig
OTU_1292	282.38	281.46	252.94	308.22	Non-sig
OTU_1599	300.10	280.98	219.93	339.32	Non-sig
OTU_1186	301.09	281.77	265.78	298.12	Higher
**Plant N niche values**					
OTU_2909	12.92	12.47	11.82	13.06	Non-sig
OTU_1017	13.06	12.47	11.54	13.30	Non-sig
OTU_2435	13.08	12.48	11.85	13.08	Higher
OTU_1118	13.02	12.47	11.62	13.28	Non-sig
OTU_1260	13.46	12.50	11.78	13.20	Higher
OTU_1189	14.39	12.53	11.10	14.14	Higher
OTU_1353	13.45	12.45	9.16	16.26	Non-sig
OTU_1151	12.50	12.45	10.50	14.46	Non-sig
OTU_1525	8.76	12.42	10.02	15.19	Lower
OTU_1376	9.64	12.43	9.94	15.16	Lower
OTU_1035	13.35	12.68	6.70	20.27	Non-sig
OTU_1308	10.02	12.49	9.55	15.86	Non-sig
OTU_1015	10.20	12.42	9.99	15.09	Non-sig
OTU_2080	10.16	12.49	10.16	15.02	Lower
OTU_1022	12.68	12.48	10.01	14.86	Non-sig
OTU_1367	12.30	12.50	10.98	14.04	Non-sig
OTU_1357	12.62	12.49	11.69	13.28	Non-sig
OTU_1862	11.43	12.52	10.28	14.98	Non-sig
OTU_1467	12.64	12.47	11.55	13.45	Non-sig
OTU_1219	12.16	12.49	11.21	13.85	Non-sig
OTU_1347	13.04	12.49	11.52	13.45	Non-sig
OTU_1162	13.83	12.50	10.83	14.53	Non-sig
OTU_1490	12.54	12.44	10.85	14.10	Non-sig
OTU_1032	11.96	12.45	10.53	14.87	Non-sig
OTU_1975	12.60	12.48	11.26	13.62	Non-sig
OTU_1292	12.80	12.49	11.34	13.57	Non-sig
OTU_1599	12.20	12.51	9.73	15.30	Non-sig
OTU_1186	12.98	12.50	11.81	13.26	Non-sig

[&]Estimated as per Guillem Salazar (2019). EcolUtils: Utilities for community ecology analysis. R package version 0.1. https://github.com/GuillemSalazar/EcolUtils.

Figure S1. Venn diagram showing number of unique and shared OTUs of total OTUs between different plant parts for individual grass species.

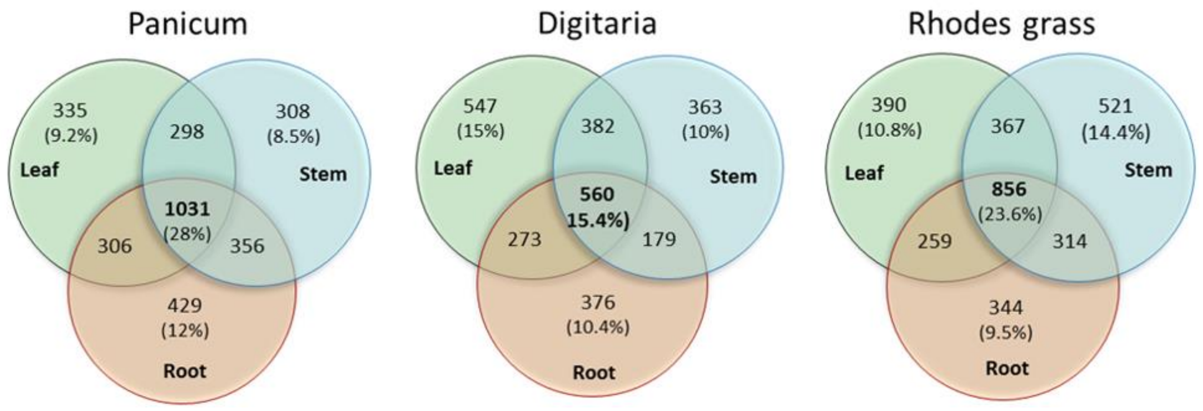

Figure S2. Relative abundances (averages of replicates) of top 17 diazotrophic (*nifH*-gene harbouring) genera accounting for >85 of total OTU abundance in different plant parts (leaf, stem and root) for the summer-active perennial grasses at Karoonda in Australia (this study) and the Switchgrass at the Kellogg Biological Station in USA (adopted from the data reported by Roley et al., 2019).

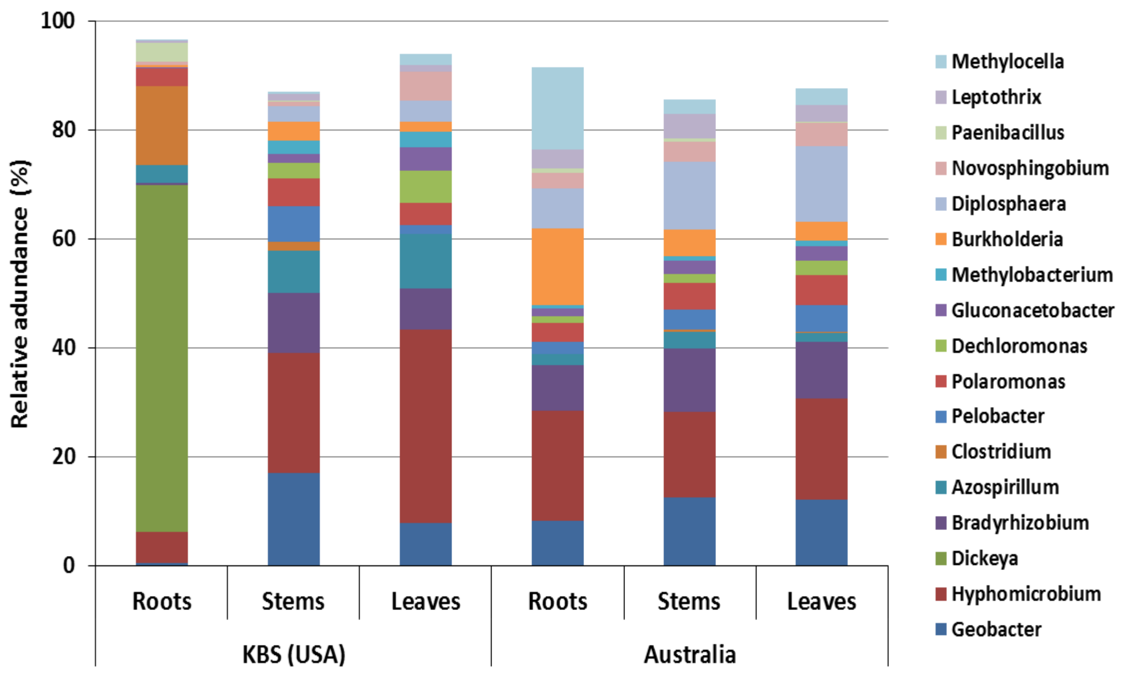

Figure S3. Relative abundances (averages of replicates) diazotrophic communities in (A) different plant parts (leaf, stem and root) and (B) above- vs. below-ground parts for the summer-active perennial grasses at Karoonda in Australia. Asterix * indicates significant differences between the different plant parts at P<0.05.

(A) Individual plant parts

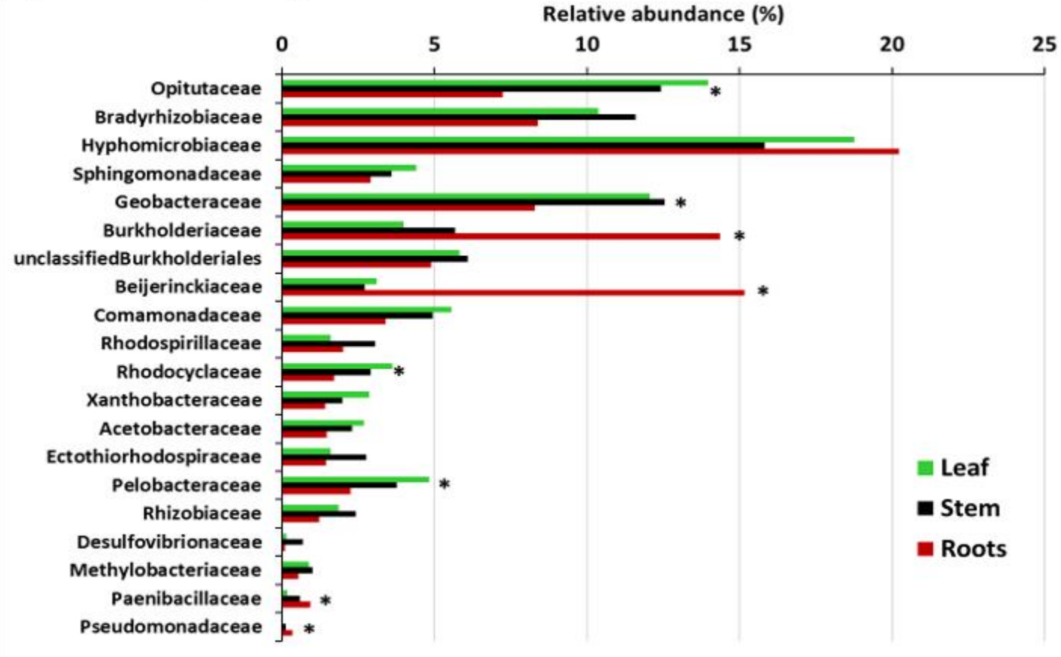

(B) Above *vs*. Below ground

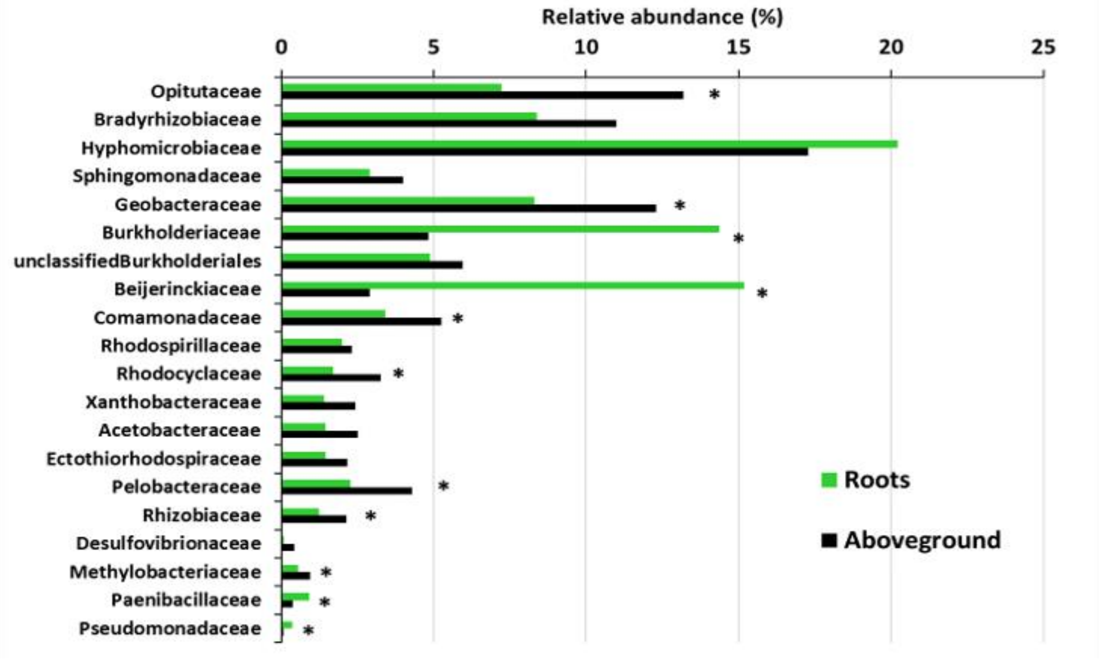

Figure S4. Diazotroph (*nifH*-gene harbouring) community network generated based on RMT-based network analysis. Circles represent nodes whose size indicates connectivity, node colour represents taxonomy at the genus level. Edges indicate co-occurrence between nodes coloured either blue for positive or red for negative. Each circular grouping is a module. Each circular grouping is a module, modules containing at least five nodes are identified by their assigned number.

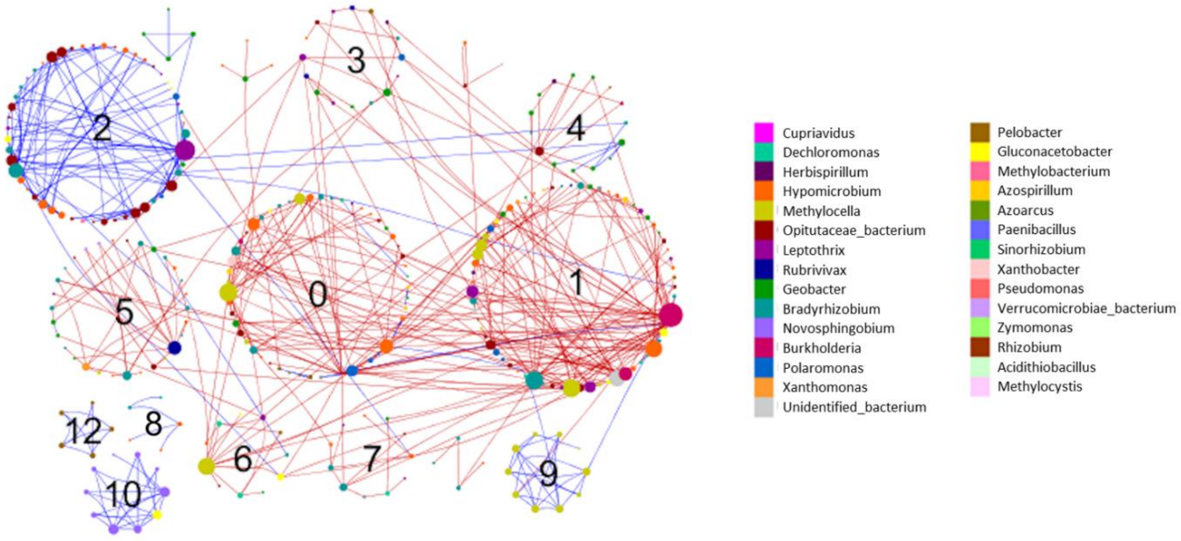

Figure S5. Topological roles of OTUs based on distribution of nodes on Zi (within module) vs. Pi (among module) connectivity scatter plots for the leaf, stem and root samples. (A) For individual plant part; each color represents an OTU from the three networks and (B) For the entire endophytic *nifH*-gene harbouring community.

(A) Individual plant parts

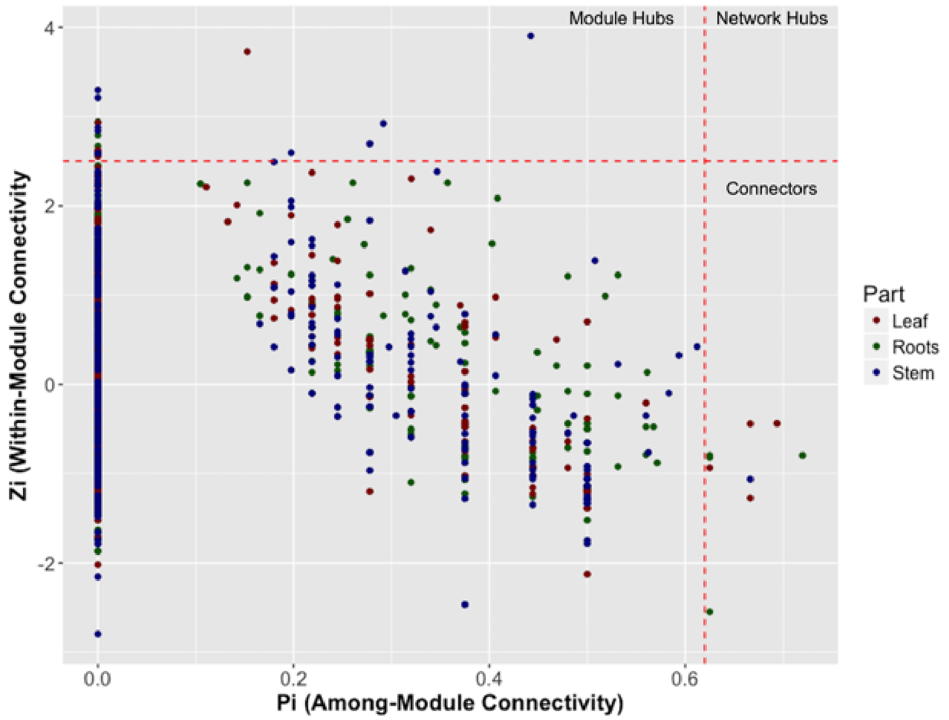

(B) All plant parts

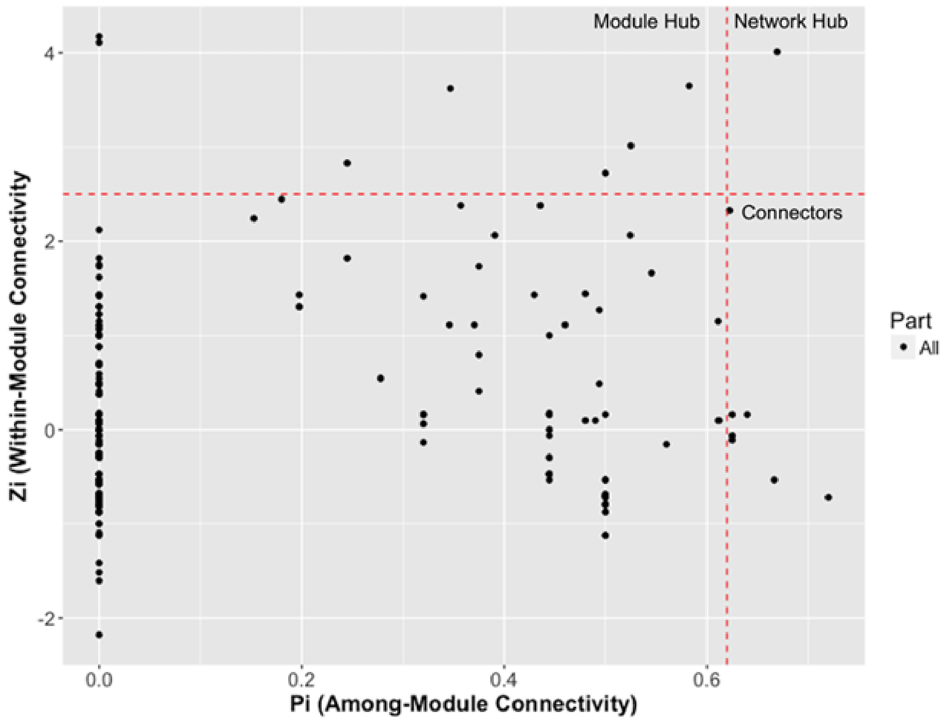

Figure S6. Pearson correlation resulting from a Mantel correlogram between the pairwise matrix of OTU niche distances and phylogenetic distances with 999 permutations. Significant correlations ($p < 0.05$, solid squares) indicate phylogenetic signal in species ecological niches.

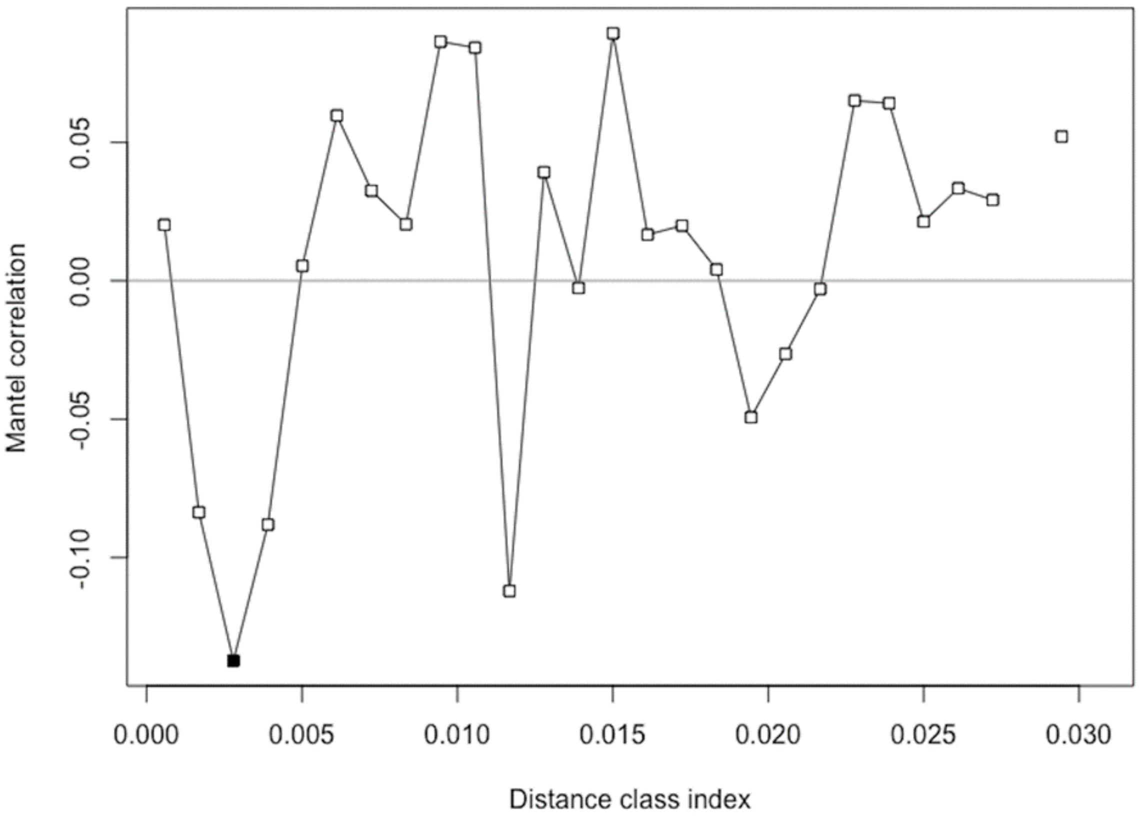

Figure S7. The net relatedness index (NRI) and nearest taxon index (NTI) for three plant parts for three plant species. Positive NRI or NTI indicates phylogenetic clustering and negative NRI or NTI indicates phylogenetic evenness.

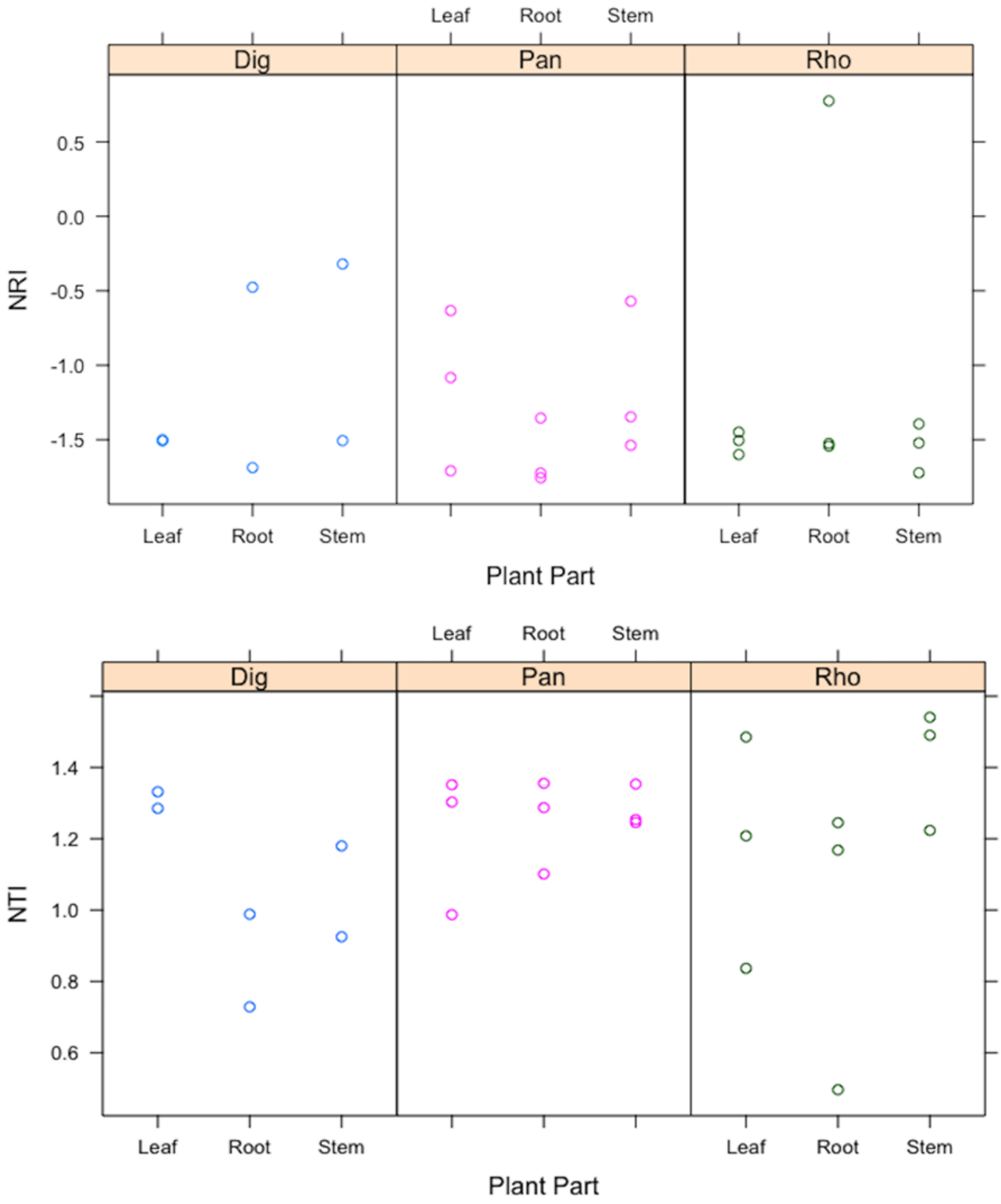

Supplement: Supplementary file 1 [file Table_1.pdf]
